# Crosstalk between SUMOylation and other post-translational modifications in breast cancer

**DOI:** 10.1186/s11658-024-00624-3

**Published:** 2024-08-10

**Authors:** Bajin Wei, Fan Yang, Luyang Yu, Cong Qiu

**Affiliations:** 1 The Department of Breast Surgery, Key Laboratory of Organ Transplantation, Key Laboratory of Combined Multi-Organ Transplantation, The First Affiliated Hospital, School of Medicine, Zhejiang University, Hangzhou, China; 2 State Key Laboratory for Diagnosis and Treatment of Infectious Diseases, National Clinical Research Center for Infectious Diseases, Collaborative Innovation Center for Diagnosis and Treatment of Infectious Diseases, Hangzhou, China; 3 MOE Laboratory of Biosystems Homeostasis & Protection, College of Life Sciences, Zijingang Campus, Zhejiang University, No. 866 Yuhangtang Road, Hangzhou, 310058 Zhejiang China; 4 Cancer Center, Zhejiang University, Hangzhou, China

**Keywords:** Breast cancer, Post-translational modifications, SUMOylation, Crosstalk

## Abstract

Breast cancer represents the most prevalent tumor type and a foremost cause of mortality among women globally. The complex pathophysiological processes of breast cancer tumorigenesis and progression are regulated by protein post-translational modifications (PTMs), which are triggered by different carcinogenic factors and signaling pathways, with small ubiquitin-like modifier (SUMOylation) emerging as a particularly pivotal player in this context. Recent studies have demonstrated that SUMOylation does not act alone, but interacts with other PTMs, such as phosphorylation, ubiquitination, acetylation, and methylation, thereby leading to the regulation of various pathological activities in breast cancer. This review explores novel and existing mechanisms of crosstalk between SUMOylation and other PTMs. Typically, SUMOylation is regulated by phosphorylation to exert feedback control, while also modulates subsequent ubiquitination, acetylation, or methylation. The crosstalk pairs in promoting or inhibiting breast cancer are protein-specific and site-specific. In mechanism, alterations in amino acid side chain charges, protein conformations, or the occupation of specific sites at specific domains or sites underlie the complex crosstalk. In summary, this review centers on elucidating the crosstalk between SUMOylation and other PTMs in breast cancer oncogenesis and progression and discuss the molecular mechanisms contributing to these interactions, offering insights into their potential applications in facilitating novel treatments for breast cancer.

## Introduction

Breast cancer is the most common cancer type worldwide, accounting for approximately 30% of cancers in women [1]. Annually, approximately 2 million women are newly diagnosed with breast cancer [2], and its global incidence has been increasing, with an annual increase of approximately 3.1%; what is worse is that this trend may continue [3, 4]. Despite notable advancements in high-quality prevention strategies, early detection, and treatment services that have led to a decline in breast cancer mortality rates, it still accounts for a substantial proportion of deaths, ranging from 15 to 30% among newly diagnosed cases [5–7]. Therefore, breast cancer remains a serious public health concern worldwide.

As a heterogeneous disease, breast cancer is commonly classified into three subtypes based on receptor expression in clinical settings: luminal estrogen receptor (ER) and progesterone receptor (PR)-positive breast cancer, human epidermal growth factor receptor 2 (HER2)-positive breast cancer, and triple-negative breast cancer (TNBC) (ER^−^, PR^−^, and HER2^−^) [8, 9]. Luminal ER and PR-positive breast cancer can be further divided into two subtypes based on the proliferation marker Ki-67: luminal A, which exhibits low Ki-67 levels, and luminal B, characterized by high Ki-67 levels [9–11]. TNBC can be divided into six categories: basal-like 1, basal-like 2, immunomodulatory, mesenchymal, mesenchymal stem cell-like, and luminal androgen receptor [12]. Notably, these breast cancer subtypes exhibit varying mortality rates, with HER2-positive breast cancer being associated with the highest mortality rate, followed by TNBC, Luminal A, and then Luminal B subtypes [13].

Breast cancer is often accompanied by two types of gene mutations: gain-of-function mutations in oncogenes and loss-of-function mutations in tumor suppressor genes. Approximately 10% of all cases are associated with genetic predisposition or family history [9]. Breast cancer susceptibility gene 1 (*BRCA1)* (located at 17q21) and *BRCA2* (at 13q13) are two important and high-penetrance tumor suppressor genes whose mutations exhibit an autosomal dominant inheritance pattern [4, 14–16]. Germline mutations in *BRCA1* or *BRCA2* contribute to approximately 15%–20% of all TNBC cases and 10%–15% of HER2-negative, hormone receptor-positive breast cancers [17]. The development of next-generation sequencing has led to the identification of more mutated genes in a series of early breast cancers, including tumor protein p53 (*TP53*) (41% of tumors), phosphatidylinositol-4,5-bisphosphate 3-kinase catalytic subunit alpha (*PIK3CA*) (30%), *MYC* (20%), phosphatase and tensin homolog (*PTEN*) (16%), cyclin D1 (*CCND1*) (16%), *ERBB2* (13%), fibroblast growth factor receptor 1 (*FGFR1*) (11%), and *GATA3* (10%) [18, 19]. This comprehensive genomic profiling has deepened insights into the molecular underpinnings of breast cancer development and potential therapeutic targets.

Although gene mutations affect specific protein sequences, the intricate functional regulation of proteins is primarily regulated by diverse post-translational modifications (PTMs). These PTMs involve chemical alterations to proteins that significantly modify their biochemical properties and are estimated to influence approximately 50% to 90% of all human proteins [20]. Each type of PTMs consists various enzymes to mediate the modification and de-modification to ensure it is in a dynamic balance. However, dysregulation of these enzymes contributes to a variety of pathologies to drive diseases. Owing to technological advancements over the past decade, several dysregulated enzymes [21–28] have been discovered contributing to imbalanced PTMs, including phosphorylation, ubiquitination, SUMOylation, neddylation, citrullination, acetylation, methylation, glycosylation, palmitoylation, succinylation, and S-Nitrosylation in breast cancer [24, 29–41]. These PTMs regulate DNA damage repair, signal transduction, immune responses, metabolic reprogramming, cell proliferation, cell cycle regulation, angiogenesis, malignant transformation, cell epithelial–mesenchymal transition (EMT) and invasion, and autophagy and apoptosis by effecting the stability, cellular localization, activity, interaction with other macromolecules, and cellular responses to different stimuli of the target substrates [20, 42–48] to play either a promotive role or a suppressing role in breast cancer.

SUMOylation, a type of ubiquitination-like modification, was described for the first time more than 25 years ago [49, 50]. It is characterized by the attachment of small ubiquitin-like modifier (SUMO) proteins to the lysine residues of target proteins [50, 51]. The SUMO family consists of three members: SUMO1–3, in which SUMO1 shares only 50% homology with SUMO2 and SUMO3, whereas SUMO2 and SUMO3 have > 97% sequence similarity [52–54]. SUMOylation is catalyzed by a cascade of three enzymes: activating enzyme (E1), conjugating enzyme (E2, UBC9), and ligating enzyme E3 [36, 55]. As one of the most dynamic modifications, six SUMO-specific protease (SENP) family proteins, namely, SENP1–3 and SENP5–7, can readily deconjugate SUMO molecules [56]. Among the SENPs, SENP1 plays a central role in deconjugating both SUMO1 and SUMO2/3 modifications in many target proteins and is therefore involved in many cellular processes [57]. Our studies, as well as other’s have demonstrated that SUMOylation plays important roles in multiple cellular processes, such as signaling transduction, gene regulation, DNA damage repair, cell death, and cell proliferation, primarily by affecting the cellular localization, stability, activity, protein–DNA, or protein–protein binding of substrates [57–66].

SUMOylation exerts critical functions in breast cancer progression. In general, SUMOylation promotes breast cancer by boosting tumor cell proliferation, migration and EMT. For example, SUMOylation of BRCA1 at K32 and K1690 has been shown to induced G0/G1 phase transition in the ER-positive breast cancer cells [67]. In addition, SUMOylation of talin at K2445 and K841 positively impacts the migration of MDA-MB-231 cells through the facilitation of focal adhesion disassembly [68]. Moreover, SUMOylation of transforming growth factor beta (TGF-β) receptor 1 (TβRI) at K389 promoted cancer cell metastasis by enhancing the interaction between TβRI and SMAD2/3, which in turn activates the TGF-β–SMAD signaling pathway and EMT [69]. However, SUMOylation of SMAD4 at K159 inhibited the TGF-β–SMAD4 signaling pathway by enhancing the interaction between SMAD4 and the transcriptional corepressor Daxx [70]. Additionally, the SUMOylation of PIN1 at K6 and K63 suppressed its oncogenic function [71]. These findings collectively indicate that SUMOylation can serve both pro-oncogenic and tumor-suppressive roles in breast cancer. Considering that SUMOylation is somewhat newly discovered, more functions and mechanisms by which SUMOylation is involved in breast cancer remains to be further investigated.

Nevertheless, breast cancer is regulated by a complex network of signaling pathways that are not controlled by only one PTM, but rather by the coordinated actions of PTM combinations. During breast cancer progression, multiple PTMs or the same PTM at different modification sites always occur on a substrate. Multiple PTMs may simultaneously or sequentially occur, which is necessary for the distinct outcomes of signaling cascades. Therefore, understanding the crosstalk between different PTMs is crucial for unraveling the molecular mechanisms and developing precise therapeutic strategies for breast cancer. Up to date, SUMOylation has been found to interact with other PTMs, in particular, with phosphorylation, ubiquitination, acetylation, and methylation in breast cancer. Therefore, in the present review, we discuss the crosstalk between SUMOylation and these four PTMs to detail the role and molecular mechanisms of each of these crosstalk pairs in regulating breast cancer oncogenesis, offering insights into their potential clinical applications in breast cancer treatment.

### Phosphorylation and SUMOylation in breast cancer

Phosphorylation is possibly the most common PTM type and has a history of more than 60 years [72–74]. In breast cancer, phosphorylation is widely involved in multiple biological processes. Therefore, it frequently interacts with other types of PTM. Unsurprisingly, there is extensive crosstalk between phosphorylation and SUMOylation in breast cancer. To date, this interaction has mainly been observed in nuclear proteins and signaling transduction through phosphorylation-directed SUMOylation, although instances of SUMOylation-directed phosphorylation have also emerged. Thus, understanding the relationship between the two PTMs is vital for clarifying breast cancer pathogenesis, drug resistance mechanisms, or new therapeutic drug development.

Given that nuclear proteins are the predominant targets of SUMOylation [75], phosphorylation-directed SUMOylation mainly occurs on such proteins. In breast cancer, phosphorylation of Krüppel-like factor 8 (KLF8), a key oncogene regulating gene transcription and breast cancer-related cellular processes, at Ser-80 is needed for SUMOylation at K67 upon DNA damage; this may be a novel mechanism promoting DNA repair and cell survival in breast cancer due to the inhibitory role of KLF8 SUMOylation on its transcription activity, functioning as a negative feedback [76]. Interestingly, this feedback is broad in the phosphorylation-SUMOylation crosstalk. For instance, phosphorylation dependent GATA1 SUMOylation inhibits its DNA binding activity [77, 78], while signal transducer and activator of transcription (STAT) 1 phosphorylation at Y701 promotes SUMOylation at K703, which then suppresses further STAT1 phosphorylation to protect cells from interferon γ (IFNγ) hypersensitivity [79]. Therefore, phosphorylation dependent SUMOylation seems like a negative feedback mechanism to avoid substrate hyperactivity. However, SUMOylation can also positively reinforce substrate activity. The phosphorylation of ERRalpha1 at Ser19 enhances its SUMOylation at K14, further promoting the transcriptional activities of ERRalpha1 by affecting its response to coactivator [80]. However, in the case of ERβ, phosphorylation at Ser6, while it does enhance SUMOylation at K4, this subsequent SUMOylation suppresses the transcriptional activity of ERβ in breast cancer cells [81]. On the other hand, if SUMOylation is depressed by phosphorylation, the situation diverges. For example, the inhibited SUMOylation of tumor suppressor p53 mediated by its phosphorylation can enhance p53 transcription activity [82]. Another instance shows that the phosphorylation-mediated inhibition of SUMOylation on the pro-inflammatory factor inhibitor of kappa B alpha (IκBα) can boost IκBα ubiquitination, accelerating degradation and promoting p65/p50 translocation [83].

The biological process of tumorgenesis is driven by signaling transduction, of which, Rac-alpha serine/threonine-protein kinase (AKT) hyperactivation is one of the most commonly observed in breast cancer, typically stemming from PTMs rather than genetic mutations in the kinase. Among these PTMs, the crosstalk between phosphorylation and SUMOylation significantly affects AKT activity. AKT can be modified by SUMO1 and SUMO2; however, irrespective of SUMO1- or SUMO2-type modification, SUMOylation promotes AKT activity, thereby regulating MCF-7 cell proliferation [84]. This feedback affects not only cell proliferation but also macrophage polarization in tumors. Enhanced AKT1 SUMOylation upon SENP3 loss resulted in AKT1 hyperphosphorylation and activation, thereby facilitating M2 polarization, breast cancer cell proliferation and metastasis [85]. Similarly, other kinases exhibit crosstalk, as seen in DDX5 where phosphorylation-dependent SUMOylation stabilizes the protein and boosts the formation of the DDX5/Drosha/DGCR8 complex, promoting microRNA-10b processing and ultimately contributing to breast cancer cell proliferation, invasion, and metastasis [86].

**Fig. 1:**
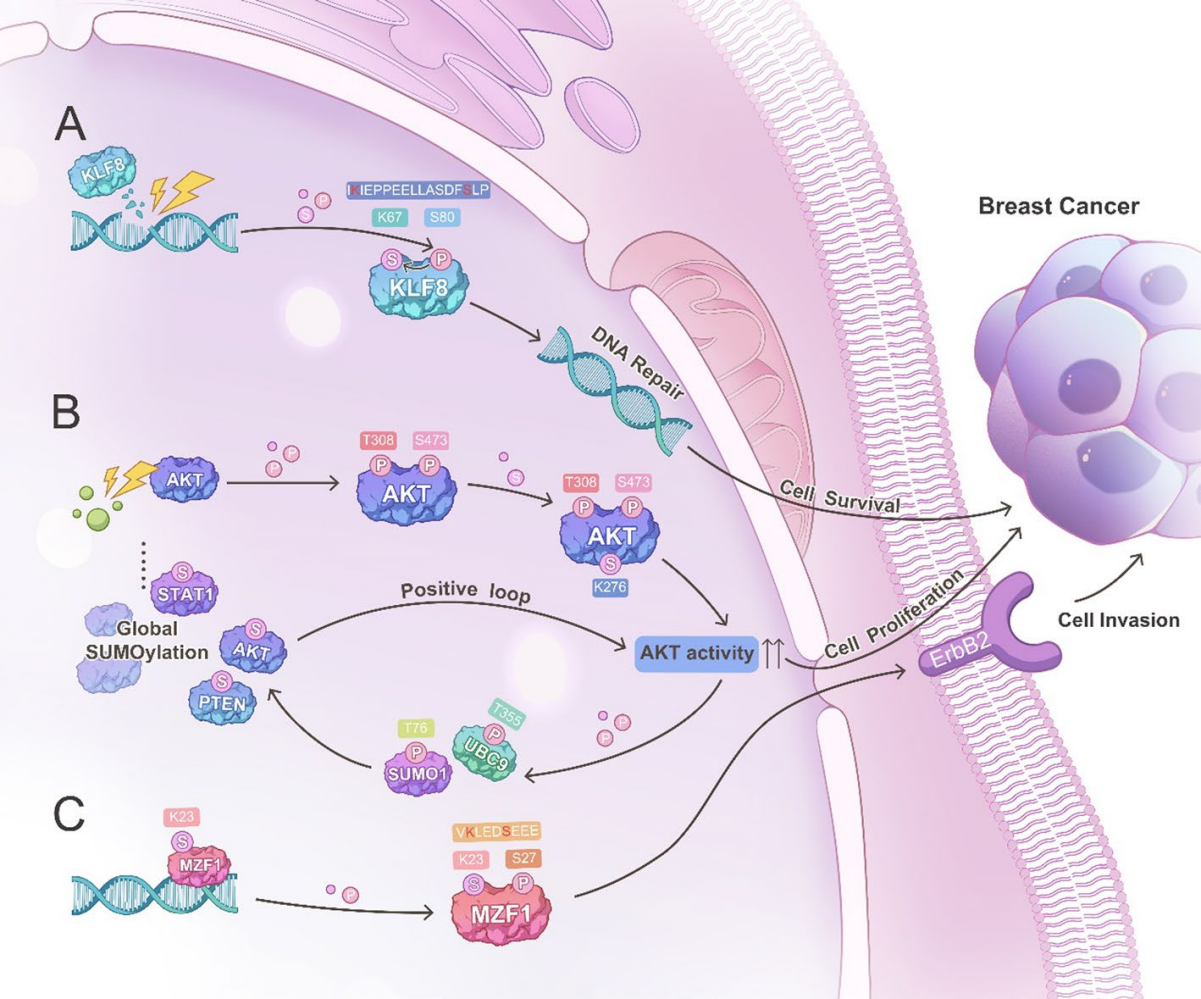
Three potential mechanisms of crosstalk between SUMOylation and phosphorylation in breast cancer. **A** Phosphorylation dependent SUMOylation of KLF8. Upon DNA damage signals, phosphorylation of KLF8 at S80 promotes KLF8 SUMOylation at K67 because of the phosphorylation dependent SUMOylation motif at K67-S80, regulating DNA damage repair and breast cancer cell survival. **B** AKT SUMOylation enhances phosphorylation of UBC9 (the only SUMOylation E2) and SUMO1 to further promote global SUMOylation. Upon pro-tumorigenic stimuli, AKT undergoes phosphorylation at the T308 and S473 sites, and subsequently mediates SUMOylation at K276, leading to enhanced AKT activity. Upregulated AKT promotes mediated the phosphorylation of UBC9 at T35 and SUMO1 at T76, thereby further leading to the enhancement of SUMOylation of multiple proteins such as AKT, STAT1, PTEN, etc. Enhanced AKT SUMOylation further promotes AKT activity, thus forming a positive feedback loop to regulate the cellular function of tumor cells and the occurrence of tumors. **C** SUMOylation dependent phosphorylation of MZF1. SUMOylation of MZF1 at K23 promotes MZF1 phosphorylation at S27, thereby further regulating the ERBB2 signaling pathway and breast cancer cell invasion

What could be the molecular mechanism behind the phosphorylation-directed SUMOylation in breast cancer? The main mechanism may be owing to the presence of a phosphorylation-dependent SUMOylation motif (PDSM) characterized by ΨKx(D/E)xxSP, where ΨKx(D/E) represents a SUMO consensus site followed by any two residues and a serine and a proline-directed phosphorylation site [77, 87, 88]. Many nuclear proteins contain the PDSM, including KLF8 (Fig. 1A), ER, heat shock transcription factor 1 (HSF1), myocyte enhancer factor 2 (MEF2), GATA1, peroxisome proliferator activated receptor gamma (PPARγ), and nuclear receptor corepressor (NCoR) [77, 89]. The phosphorylation of the serine or proline residues of this motif provides the essential negative charge, enabling interaction with the basic residues of UBC9 or SUMOs, thereby enhancing SUMO conjugation [81, 90]. Similar to PDSM, the negative charge-dependent SUMOylation motif (NDSM) (ΨKXEXXEEEE) also contains the ΨKxE consensus motif, followed by at least two acidic residues localized < 10 residues away from the C-terminal end of the target lysine residue [90]. In addition to consensus covalent SUMOylation, the nonconsensus SUMO conjugation motif is also phosphorylation-dependent, where the consensus D/E residue is substituted for a serine residue, and whose phosphorylation provides the negative charge for nearby SUMOylation, such as ER [81]. This extended PDSM offers a valuable signature for predicting SUMO substrates that are regulated by protein kinases. Indeed, for PR, the PDSM is absent [87], and this may be why there is always controversial over whether there is phosphorylation dependent SUMOylation on PR. However, for proteins such as AKT, which also do not contain these motifs, its SUMOylation can also been modified by altering the characteristics of SUMO-related enzymes or molecules. Phosphorylation-dependent AKT SUMOylation could occur because AKT phosphorylation increases its own activity and directly phosphorylates UBC9 at Thr35 and SUMO1 at Thr76, fostering UBC9 thioester bond formation and SUMO1 stabilization, thereby amplifying AKT SUMOylation and creating a positive feedback loop. This heightened AKT-induced phosphorylation of UBC9 and SUMO1 also impacts the SUMOylation of other proteins, such as PTEN, further governing cellular processes in breast cancer in breast cancer (Fig. 1B) [91]. However, for cases where phosphorylation inhibits SUMOylation, the precise mechanism remains unclear. One possibility is that substrate phosphorylation inhibits its binding to SUMO ligase, supported by the p53 case where SUMOylation of p53 is inhibited by site-specific phosphorylation, which reduces the binding of p53 to UBC9 [92]. Although SUMOylation of c-Jun, ETS domain-containing protein Elk1 (ELK1), and promyelocytic leukemia (PML) are also repressed by phosphorylation, the crosstalk mechanism is still unknown, possibly due to a conformational change caused by phosphorylation that makes the SUMOylation site exposed to enable more rapid cleavage by SUMO proteases [93–96]. Taken together, phosphorylation-directed SUMOylation through PDSM or NDSM is the core mechanism driving the crosstalk between these two PTMs.

In addition to phosphorylation-directed SUMOylation, there is also a new crosstalk called “SUMO-directed phosphorylation” in breast cancer. This process involves the poly-SUMOylation of myeloid zinc finger-1 (MZF1) at K23 directs MZF1 phosphorylation at S27 to further mediate invasive ERBB2 signaling in breast tumors (Fig. 1C) [97]. This crosstalk through a mechanism where SUMOylation at K23 opens up and exposes the S27, which otherwise is masked and not approachable for phosphorylation.

In conclusion, the phosphorylation of substrate proteins may have either positive or negative effects on SUMOylation in breast cancer, with the majority of studies suggesting a positive effect. Given that SUMOylation regulates the subcellular localization, protein stability, and protein–protein/DNA binding of substrate proteins, phosphorylation directed SUMOylation may play a feedback role through these ways to prevent substrate hyperactivity and cellular homeostasis or work synergistically with each other to enhance substrate activity. Through these mechanisms, phosphorylation-dependent SUMOylation can fundamentally alter the biological properties of substrate proteins, contributing further to breast cancer progression by influencing tumor cell proliferation, metastasis, and mitochondrial function.

### SUMOylation and ubiquitination in breast *cancer*

Ubiquitination is a multi-step process catalyzed by ubiquitin-activating enzymes (E1), ubiquitin-conjugating enzymes (E2), and ubiquitin ligases (E3), which is similarly to SUMOylation [43]. Ubiquitination either promotes or suppress breast cancer. There is substantial evidence demonstrating that SUMOylation directly influences ubiquitination, which we will discuss by examining three aspects: SUMOylation-induced ubiquitination, SUMOylation-repressed ubiquitination, and SUMO type-specific effects on ubiquitination.

**Fig. 2:**
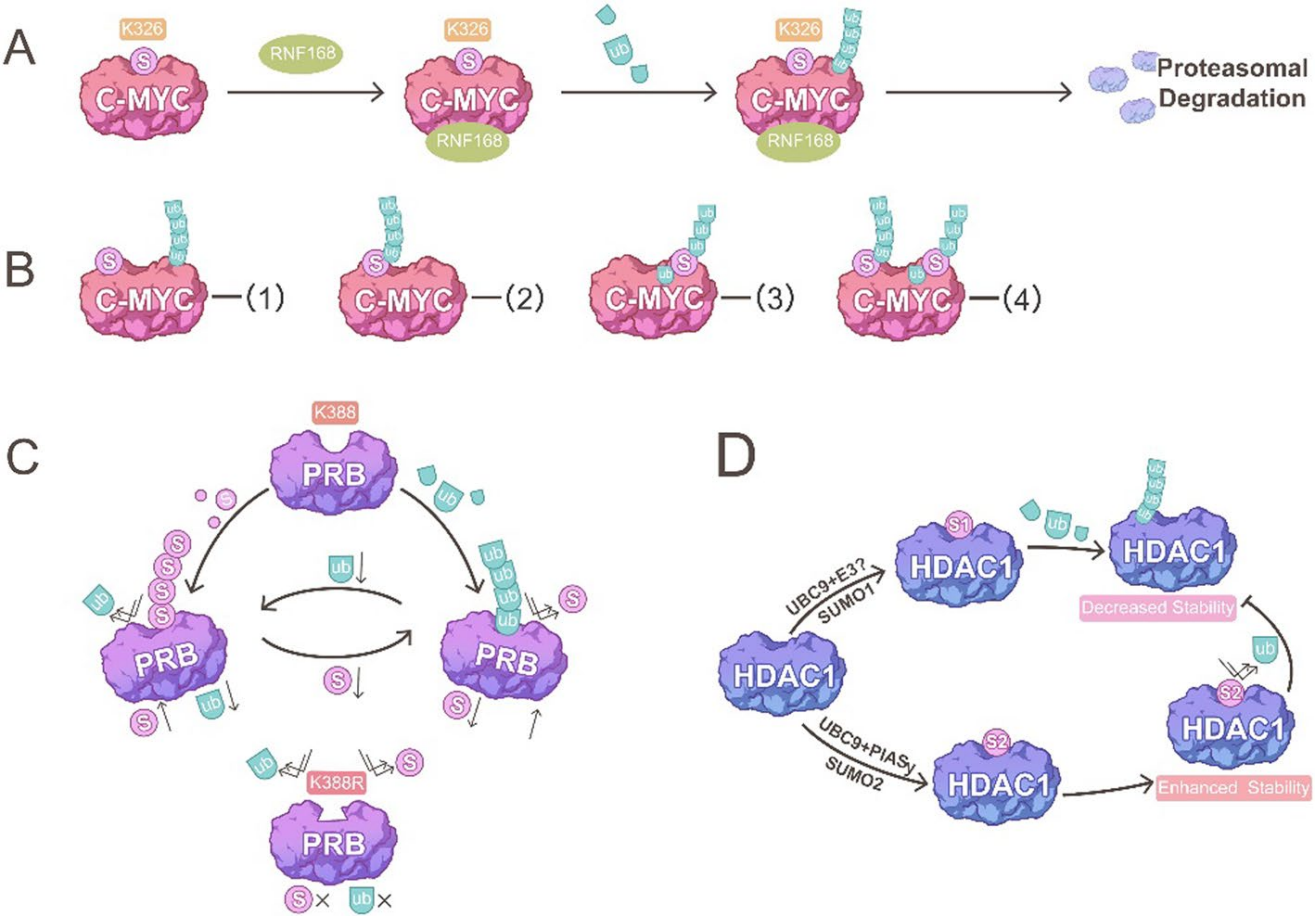
Main mechanisms of the crosstalk between SUMOylation and ubiquitination in breast cancer. **A** SUMOylation of c-MYC promotes its ubiquitination and degradation. SUMOylated c-MYC at K326 increases recruitment of RNF168 (ubiquitination E3 ligase) to enhance c-MYC ubiquitination. **B** Potential mechanisms of crosstalk between SUMOylation and ubiquitination at c-MYC. (1) SUMOylation and ubiquitination at different sites. (2) Ubiquitination occurs on SUMO molecules. The SUMOylation of c-MYC induces the binding of ubiquitin molecules on SUMO, leading to c-MYC proteasome degradation. (3) SUMOylation occurs on ubiquitin molecules, followed by further ubiquitination on SUMO. (4) A mixture of form (2), (3), and (4). **C** Progesterone receptor isoform B (PRB) SUMOylation competitively inhibits its ubiquitination. The ubiquitination site and the SUMOylation modification site are both located at the K388 of PRB. Once the K388 is mutated, neither SUMOylation nor ubiquitination of PRB can occur. **D** Different SUMOylation types of HDAC1 regulate different ubiquitination. In breast cancer tissue, SUMO2-type modification mediated by E3 ligase PIASy inhibits HDAC1 ubiquitination, thus improving HDAC1 stability. However, the SUMO1-type modification promotes HDAC1 ubiquitination, thereby reducing its protein stability

SUMOylation promotes ubiquitination to play a role in breast cancer suppression. For example, the SUMOylation of forkhead box M1 (FOXM1) at multiple sites—K201, K218, K460, K478, and K495—which facilitates ring finger protein (RNF) 168 recruitment, leading to FOXM1 ubiquitination and degradation, thereby inhibiting MCF-7 cell proliferation and mitotic progression [98, 99]; this may suppress breast cancer progression, metastasis, and genotoxic agent responses [100–104]. The proteasome degradation pathway of c-MYC, a frequently overexpressed oncogene in breast cancer, may also depend on SUMOylation; its SUMOylation at K326 results in its subsequent ubiquitylation and degradation by the proteasome (Fig. 2A) [105, 106]. This finding has been further confirmed by another study showing that SENP1, the major deSUMOylase often overexpressed in breast cancer tissues, leading to c-MYC deSUMOylation and the subsequent decrease in c-MYC polyubiquitination; this results in high c-MYC expression, leading to breast cancer cell proliferation and transformation [107].

Several reasons may contribute to SUMOylation-dependent ubiquitination: 1) Similar to phosphorylation, these proteins may contain a SUMOylation-dependent ubiquitination motif to ensure SUMOylated proteins are better substrates than non-SUMOylated proteins (Fig. 2B, module 1). To support this, a study has reported that SUMOylation promotes an MYC mutant that cannot target FBW7 to be a FBW7 substrate [107]. 2) Substrates may be co-modified by both SUMO and ubiquitin to form a SUMO–ubiquitin chain because the ubiquitination of SUMO and SUMOylation at multiple lysine residues of ubiquitination have been identified [23, 108] (Fig. 2B, module 2–4). Evidence exists where MYC can be co-modified by both SUMO and ubiquitin, and SENP1 can stabilize MYC by removing ubiquitination via deSUMOylation [109]. Moreover, a single ubiquitin attached to MYC was identified by overexpressing SENP1 without proteasome inhibition; this indicates that SUMOylation occurs on a single ubiquitin molecule [109]. 3) SUMOylation may affect the stability or activity of the ubiquitin ligases, thereby promoting global ubiquitination [105, 109–112]. A similar mechanism has been observed during DNA double-strand break repair. Both SUMOylated RNF168 and HECT and RLD domain containing E3 ubiquitin protein ligase 2 (HERC2) enhance their association with RNF8, leading to the formation of an active UBC13-RNF8 complex that facilitates ubiquitin chain formation at the site of DNA damage [113]. 4) Another mechanism may involve the SUMO-targeted E3 ubiquitin ligase (STUbL) RNF4 [113]. The N-terminus of RNF4 contains four SUMO-interacting motifs (SIMs, SIM1-4) for recognizing poly-SUMOylated substrates. Among these motifs, SIM2 and SIM3 play a significant role in binding to SUMO-2 chains while SIM1 and SIM4 have a minor role [114, 115]. Binding to poly-SUMO chains induces the dimerization of the C-terminal RING domains of RNF4, which stabilizes the E2-ubiquitin thioester bond and subsequently catalyzes poly-ubiquitination of the substrates [113, 116–119]. Typically, this type of poly-ubiquitination results in proteasome-mediated degradation [120, 121]. In addition, RNF4 is also implicated in recruiting proteasome components to indirectly promote ubiquitin conjugation and proteasomal degradation [117]. However, additional studies are warranted to reveal the in-depth molecular mechanism of the crosstalk in breast cancer.

On the other hand, SUMOylation predominantly inhibits substrate ubiquitination and proteasomal degradation pathways by competing for the same lysine residues. Breast cancer-associated gene 2 (BCA2), an E3 SUMO ligase for IκBα in breast cancer cells, promotes IκBα SUMOylation, thereby preventing its ubiquitination for proteasomal degradation and boosting breast cancer cell proliferation and migration [122]; similarly, K379 of delta-lactoferrin (DLf), which can be either ubiquitinated or SUMOylated, is a key site for controlling DLf stability. SUMOylation competes with ubiquitination and protects DLf degradation by positively regulating its stability [123]. In progesterone receptor isoform B (PRB), both ubiquitination and SUMOylation occur at K388, and reduced SUMOylation accelerates PRB ubiquitination, leading to a decrease in T47D cell proliferation [124]. Mutations in the K388 SUMOylation site of PRB hinder progesterone-dependent PR degradation, indicating that K388 is a dual SUMOylation and ubiquitination site; when the conjugation site is mutated, neither modification can take place (Fig. 2C) [87]. However, there are cases where mutations in SUMOylation sites enhance ubiquitination. For example, SUMOylation at Lys-2806 of zinc finger homeobox 3 (ZFHX3) enhances the stability of ZFHX3 by interfering with its ubiquitination and proteasomal degradation, while the ZFHX3 K2806R mutant decreases its protein stability, further suppressing breast cancer growth [125]. The estrogen-induced SUMOylation of pescadillo ribosomal biogenesis factor 1 (PES1) stabilizes PES1 by inhibiting its ubiquitination, but mutation of K517R promotes the PES1 ubiquitin–proteasome pathway, thereby suppressing breast cancer cell proliferation and tumor growth [126]. These proteins may be ubiquitinated at residues other than the main SUMOylation sites.

In addition, the interaction between SUMOylation and ubiquitination depends on SUMO type. While SUMO1-type SUMOylation of histone deacetylase (HDAC) 1 promotes its ubiquitination and degradation, SUMO2-type SUMOylation of HDAC1 enhances its protein stability. This selective SUMOylation may be mediated by specific SUMO E3 ligases in specific cellular environments, further leading to ubiquitination regulation. Protein inhibitor of activated STAT 4 (PIASy), overexpressed in breast cancer cells, selectively promotes the conjugation of HDAC1 to SUMO2 (Fig. 2D) [127]. However, further investigation is necessary to elucidate the mechanisms underlying how different SUMOylation types differently affect ubiquitination.

Overall, SUMOylation plays an important role in regulating ubiquitination, either enhancing or repressing it. Regardless of the direction, SUMOylation-regulated ubiquitination constitutes a critical mechanism in breast progression. Targeting this regulatory mechanism presents a potential novel therapeutic strategy.

### SUMOylation and acetylation in breast *cancer*

**Fig. 3:**
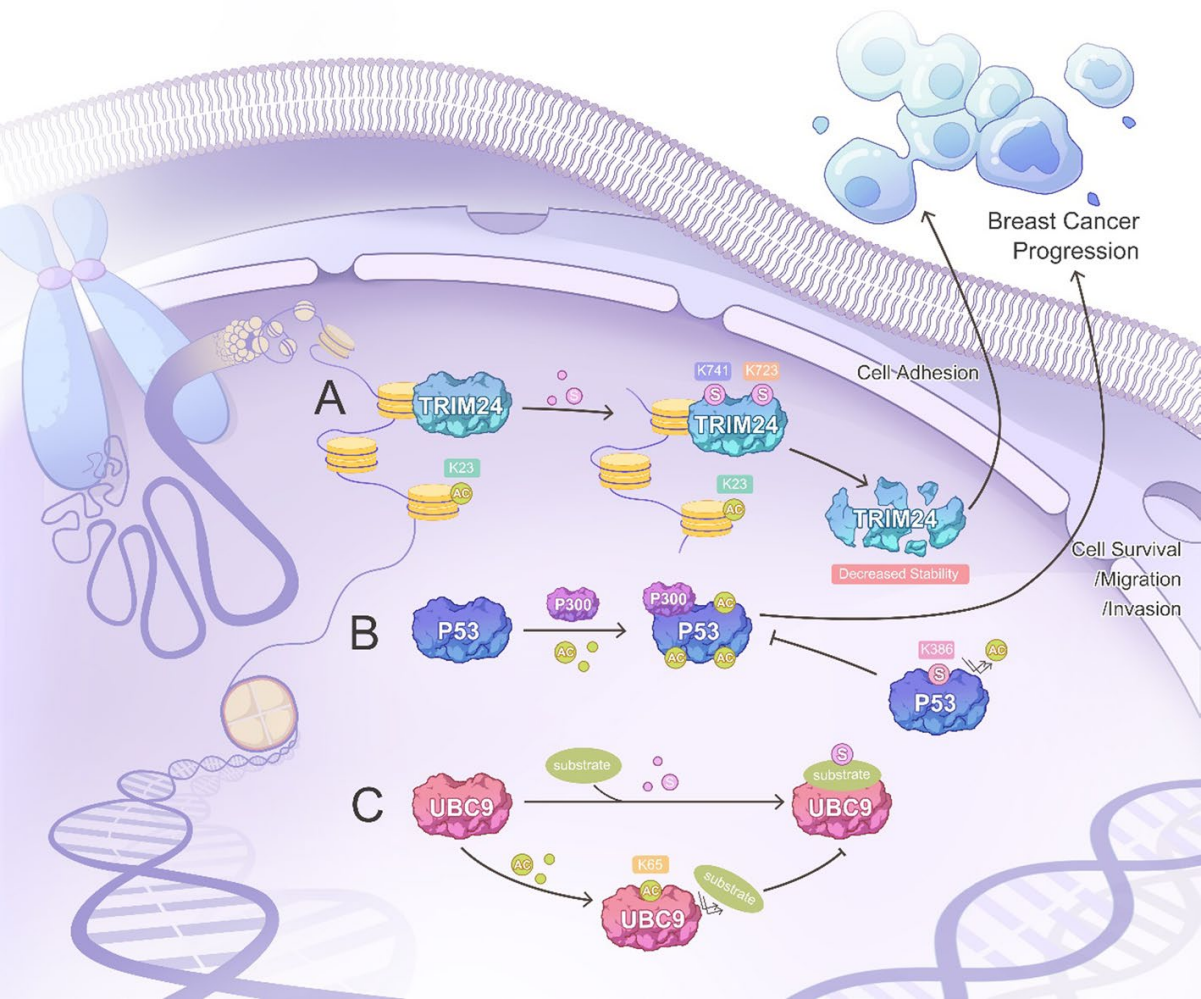
Crosstalk between SUMOylation and acetylation in breast cancer. **A** TRIM24 binds to chromatin and recognizes histone 3 with non-methylation at K4 and acetylation at K23 (H3K4me0/K23ac). Subsequently, TRIM24 undergoes SUMOylation at K723 and K741, leading to a decrease in TRIM24 stability. **B** SUMOylation of non-histone protein p53 inhibits its acetylation. p53 can be acetylated at multiple lysine sites under the mediation of P300. However, once the K386 of p53 is SUMOylated, it can inhibit p53 acetylation, thus affecting the breast cancer progression. Since the K386 is also one of the acetylation sites of P53, the SUMOylation at this site may have a competitive inhibitory effect on the acetylation at the same site. **C** Acetylation regulates SUMOylation by UBC9. Acetylation of UBC9 at K65 inhibits the binding of UBC9 to substrate proteins, thereby inhibiting systemic SUMOylation

Acetylation is a reversible process mediated by lysine acetyltransferases and deacetylases for adding and removing the acetyl group from the side chain of lysine, respectively. Both histone and non-histone proteins are substrates of acetylation. Canonical acetylation occurs in histone proteins, where modifications play an essential role in breast cancer development and prognosis. Dysregulated deacetylation promotes cancer cell proliferation, cell cycle arrest, abnormal cell death, immune destruction, immune evasion, migration invasion, and metastasis [27, 128]. Interestingly, histone acetylation has crosstalk with non-histone protein SUMOylation. A notable example involves tripartite motif-containing protein 24 (TRIM24), a histone reader aberrantly expressed in breast cancer. In that study, researchers observed that the association of chromatin with TRIM24 leads to TRIM24 SUMOylation at lysine residues 723 and 741, which depends on the acetylated lysine 23 of histone H3, further promoting cell adhesion to extracellular matrix proteins (Fig. 3A) [129]. This interaction may be an important mechanism to explore the downstream functions that regulate specific genes implicated in breast cancer [129]. Besides it, histones themselves serve as substrates for SUMOylation, regulating multiple cellular process, such as gene regulation, chromatin condensation, p300-mediated transcription, double-strand break repair, and Set3-histone-deacetylase complex-mediated transcriptional regulation [130–132]. Therefore, it is not surprising that SUMOylation has crosstalk with acetylation on histone proteins. For example, SUMOylation of histone H4 at K12 inhibits H4 tail acetylation mediated by the acetyltransferase p300, indicating a negative crosstalk between histone SUMOylation and acetylation [131]. However, the role of this crosstalk in breast cancer progression remains unexplored.

Non-histone proteins are also subject to acetylation, a process referred to as non-canonical acetylation. In breast cancer, this type of acetylation effects metastasis, cancer cells proliferation, and the sensitivity of tumor cells to anti-tumor therapy, by regulating the functions of target proteins, such as Twist, RelA/P65, oncogene nuclear receptor coactivator amplified in breast cancer 1 (AIB1), homeobox B13 (HOXB13) [20]. In essence, the crosstalk between SUMOylation and acetylation mainly occurs competitively on non-histone proteins. For instance, SUMOylation of the tumor suppressor p53 at K386 inhibits its acetylation (Fig. 3B) [133], and a decrease in p53 acetylation is known to promote breast cancer development [134–136]. Another illustrative example involves DLf, when it is acetylated at K13, this decreases its SUMOylation and enhances the transcriptional activity of DLf, thereby may possess anti-breast cancer activity [123, 137]. A similar interplay can take place among transcription co-factors. When KRAB domain-associated protein 1 (KAP1) undergoes SUMOylation, it leads to decreased H3-K9 and H3-K14 acetylation and enhanced H3-K9 methylation at the p21 promoter, thereby regulating Dox-induced p21 expression, desensitizing MCF-7 cells to Dox-elicited cell death [138]. Moreover, acetylation extends its influence to global SUMOylation by regulating UBC9, the only E2 of SUMOylation. The acetylation of UBC9 at K65 attenuates the binding of UBC9 to substrates, decreasing overall SUMOylation (Fig. 3C) [139, 140]. The dynamic switch between deacetylation and SUMOylation may represent a novel mechanism underlying breast cancer progression.

The possible mechanism involves the crosstalk is that the conjugation site of SUMOylation and acetylation are the same. What can support it is that K386 is the competitive conjugation site for both acetylation and SUMOylation of p53 and K13 is the competitive conjugation site for both acetylation and SUMOylation of DLf [82, 123]. Another possible mechanism may involve the deacetylase, such as HDAC4, which has been considered as an E3 of SUMOylation of several proteins in breast cancer progression, such as silent information regulator 1 (SIRT1), IκBα, androgen receptor, and hypermethylated in cancer 1 (HIC1) [141–144]. This suggests that HDAC4 may mediate deacetylation/SUMOylation switch simultaneously. Additionally, it is intriguing to note that acetylation can neutralize the positive charge of lysine as well as SUMO surface, thereby preventing SUMO from binding to the negatively charged residues of SUMO-interaction motifs, which contain a hydrophobic residue core that binds to the hydrophobic pocket on the SUMO for non-covalent binding [145]. Therefore, it is plausible that acetylation might also inhibit SUMOylation in breast cancer through a comparable charge neutralization mechanism.

### SUMOylation and protein methylation in breast *cancer*

Protein methylation is a process of transferring the methyl group from s-adenosyl methionine to the side chains of target protein amino acid residues, mediated by methyltransferases. The methylation at lysine and arginine residues of substrates is the prominent and universal types of methylation in breast cancer. In general, protein methylation promotes breast cancer progression by activating oncogenic signaling pathways, facilitates breast cancer cells EMT and migration by activating oncogenic genes or repress antitumor ability of tumor suppressor proteins, such as p53. Methylation at residue K370 on p53 represses its antitumor ability by inhibiting p53-mediated cancer cell apoptosis, while methylation at K382 represses its transcriptional activity [146, 147]. Interestingly, as mentioned above, p53 also can be SUMOylated. However, further study is required to analyze whether the methylation of p53 has a crosstalk with SUMOylation in breast cancer.

In fact, the crosstalk between protein methylation and SUMOylation is frequently observed in the regulation of methyltransferases or SUMO ligases themselves, rather than just oncogenes or tumor suppressors. For example, UBC9 can promote the SUMOylation of PR-Set7, a chromatin-modifying enzyme that specifically monomethylates lysine 20 of histone H4 (H4K20me1), to further decrease the expression of downstream genes mediated by PR-Set7, potentially in response to DNA damage in breast cancer [148]. Given that H4K20me1 has been reported to be associated with gene bodies, promoters, and enhancers, the repressive role mediated by SUMOylated PR-Set7 could be attributed to altered methylation at histone H4's K20 residue. On the other hand, methylation can also have an impact on SUMOylation. A case in point is the SUMO1 activating enzyme subunit 1 (SAE1), a SUMOylation enzyme; levels of DNA methylation at the SAE1 gene site (cg14042711) are negatively correlated with levels of SAE1 expression and global SUMOylation, indicating the regulatory role of methylation in SUMOylation in breast cancer [149]. Intriguingly, the AKT SUMOylation is mediated by SAE1 [150, 151], suggesting that SAE1 methylation may suppress cancer cell proliferation by reducing AKT SUMOylation.

In conclusion, unlike other types of protein modifications where crosstalk often takes place on the same substrate, the interaction between methylation and SUMOylation typically manifests through regulation of the enzymes involved in these processes, rather than direct modification of a shared substrate. This distinctive pattern may stem from the fact that protein methylation can take place at multiple amino acid residue side chains, whereas ubiquitination and acetylation, like SUMOylation, predominantly occur on lysine residues. This inherent difference allows for an indirect yet significant interplay between methylation and SUMOylation pathways.

## Conclusion and perspectives

Breast cancer is the most prevalent malignant tumor and a leading cause of mortality among women worldwide. The progression of breast cancer is intricately governed by various protein PTMs, which are modulated by an array of cytokines, drugs, tumor microenvironments, genetic factors, and signaling pathways. However, clinical trials on PTM in breast cancer are primarily focus on protein acetylation, and most of which are only in their initial stages [20]. Recent advances in omics technologies, such as mass spectrometry, high-throughput sequencing, and bioinformatics, have facilitated the identification of new PTMs and helped reveal the mechanisms by which they regulate breast cancer progression [21, 23, 48]. SUMOylation, a recently identified modification, plays an important regulatory role in regulating cancer cell proliferation, migration, and metastasis. Notably, in the progression of breast cancer, SUMOylation frequently forms intricate networks with other PTMs, leading to complex interactions. In this review, we highlighted the crosstalk between SUMOylation and other PTMs, including phosphorylation, ubiquitination, acetylation, and methylation, in breast cancer. Generally, SUMOylation has a crosstalk with these PTMs, which together regulate the various stages of breast cancer development (Table 1). These pairs of crosstalk often serve as either redundant or negative feedback regulators to maintain cellular homeostasis; however, any dysregulation can disrupt this equilibrium and drive breast cancer progression. In the crosstalk network, SUMOylation plays a central role, providing feedback regulation to phosphorylation while also influencing subsequent ubiquitination, acetylation, and methylation. Mechanistically, changes in amino acid side chain charge distribution, conformational shifts, or the occupancy of specific sites due to post-translational modifications constitute the fundamental mechanisms driving crosstalk, with unique sequences like PDSM or specific modification sites serving as the mediators for such interactions. This comprehensive review may widen our current understanding of the relationship between SUMOylation and other PTMs in breast cancer and provide new perspectives for breast cancer treatment. However, future studies are required to address several important questions that are still unanswered.

**Table 1:** The role of crosstalk between SUMOylation and other PTMs in breast cancer

Type of other PTMs	Protein	Cell type	Regulated phenotype (in vitro)	Animal model	Regulated phenotype (in vivo)	Ref
Phosphorylation	KLF8	MDA-MB-231	DNA repair Cell survival	/	/	76
Phosphorylation	ERRα1	MCF-7	Transcription activity	/	/	80
Phosphorylation	ERβ	Hs578t MCF-7	Transcription activity	/	/	81
Phosphorylation	AKT	MCF-7	Cell proliferation	/	/	84
Phosphorylation	AKT	Macrophage	AKT activity	Xenograft (Py8119 Cell)	Tumor growth Metastasis	85
Phosphorylation	DDX5	MCF-7 MDA-MB-231	Proliferation Invasion Metastasis	Xenograft (MCF-7 cell)	Tumor growth Invasion Metastasis	86
Phosphorylation	MZF1	MCF-7	Invasion	/	/	97
Ubiquitination	FOXM1	MCF-7	Cell proliferation Mitotic progression	/	/	98–99
Ubiquitination	c-MYC	T47D MDA-MB-231 SUM159	Cell proliferation Transformation	/	/	107
Ubiquitination	IκBα	MCF-7 MDA-MB-231 MCF-12F	Cell proliferation Cell migration	/	/	122
Ubiquitination	PRB	T47D	Cell proliferation	/	/	124
Ubiquitination	ZFHX3	MDA-MB-231	Cell proliferation	Xenograft (MDA-MB-231 Cell)	Tumor growth	125
Ubiquitination	PES1	MCF-7 T47D	Cell proliferation	Xenograft (MCF-7 Cell)	Tumor growth	126
Acetylation	TRIM24	MCF-7	Cell adhesion	/	/	129
Acetylation	p53	MCF-7 MDA-MB-231	Cell survival Cell migration Cell Invasion	Xenograft (ZR-75–30/MDA-MB-231 Cell)	Tumor growth	133–136
Acetylation	KAP1	MCF-7	Cell survival	/	/	138

SUMOylation is a dynamic process, in which deSUMOylation is mediated by the SENP family proteins, while SUMOylation is primarily facilitated by various SUMOylation E3 ligases [36, 55, 56]. Notably, the crucial SUMO protease SENP1 can be upregulated during tumor development and progression [57, 107], possibly due to its transcriptional regulation by HIF1α, which is significantly upregulated by the tumor microenvironment [152–157]. In addition, SUMOylation E3 ligases like BCA2 also highly expressed in breast cancer [122, 158]. These alterations in the enzymes of deSUMOylation and SUMOylation collectively disrupt SUMOylation homeostasis during breast cancer progression. SUMOylation predominantly targets nuclear proteins, and thus, the interplay between SUMOylation and other PTMs is largely observed in transcription factors, transcriptional co-regulators, and nuclear receptors. However, our previous studies have found that SUMOylation also has dominant role in regulating membrane protein, such as FGFR1 [63], and mitochondria proteins like fission protein 1 (FIS1) [65]. Of significance, these two proteins are instrumental in breast cancer progression due to their critical roles in regulating EMT and mitochondrial function, respectively. Moreover, both FGFR1 and FIS1 exhibit additional PTMs beyond SUMOylation, including phosphorylation and ubiquitination [63, 159–161]. Therefore, it is worth further investigations to reveal the crosstalk of SUMOylation and other PTMs in these non-nuclear proteins in breast cancer. Such investigations could potentially uncover novel regulatory mechanisms and contribute significantly to our understanding of breast cancer development and progression.

In addition to PTMs mentioned above, some other rare PTMs have also been reported in breast cancer. Neddylation is another type of ubiquitination-like modification that involves covalent conjugation of neural precursor cell-expressed developmentally downregulated 8 (NEDD8) to a lysine residue in the target protein [162]. Studies have documented elevated levels of neddylation in breast cancer on various targets, such as p53, Smurfl, PTEN, murine double minute 2 (MDM2), BCA3, and TGF-β II [163–169]. Given the similarities between neddylation and SUMOylation, studies have reported the crosstalk between neddylation and SUMOylation. As an example, the SUMOylation of ribosomal protein L11 (RPL11) negatively modulates the conjugation of NEDD8 to RPL11 and promotes RPL11 translocation outside the nucleoli [170]. However, to date, no studies have specifically reported the crosstalk between SUMOylation and neddylation in breast cancer. Based on the interaction between SUMOylation and ubiquitination, it is plausible to hypothesize that SUMOylation might primarily suppress neddylation, thereby regulating substrate protein localization or activity and contributing to breast cancer tumorigenesis. Furthermore, protein glycosylation has been shown to play an oncogenic role in breast cancer by promoting proliferation and metastasis of cancer cells, inhibiting the sensitivity of tumor cells to anti-tumor therapy, and altering the immune microenvironment and antitumor immune response [20]. In addition, citrullination and palmitoylation have also been reported in breast cancer. Citrullination regulates epidermal growth factor (EGF)- phosphatidylinositol 3-kinase (PI3K) signaling, nuclear localization, and TGF-β signaling, further mediating gene transcription, cell proliferation, cell invasion and migration, and cancer cell EMT in breast cancer tumorigenesis and progression [30, 45]. Palmitoylation of cluster of differentiation (CD) 44 decreases its interaction with migratory binding partner ezrin, therefore inhibiting breast cancer cell migration [171]. Nevertheless, the crosstalk between SUMOylation and these three PTM type has not been revealed in breast cancer.

Because of the central role of SUMOylation in the crosstalk, some inhibitors targeting SUMOylation in breast cancer have been explored. However, these methods are primarily conducted by regulating enzymes that affect SUMOylation, including the SAE1/2 and the unique E2 UBC9 [172–175]. Although these inhibitors play crucial anticancer roles in breast cancer cell lines, including MDA-MB-231, MCF-7, and BT474, by accelerating autophagy-dependent cancer cell death or repressing cell migration and invasion [176, 177], it should be noted that targeting these enzymes can alter global SUMOylation patterns. The specific effects of such interventions therefore require further clarification. Indeed, although the majority of SUMOylation events may facilitate breast cancer tumorigenesis and progression through accelerating cell cycle transitions and promoting EMT and tumor cell migration, a subset of SUMOylation processes function as tumor suppressors. For example, the effects of SENP1- and SENP2-mediated deSUMOylation on tumor development are different, although both can suppress global SUMOylation [42]. Another example involves AKT and c-MYC, which are both deSUMOylated by SENP1 [107, 178], suggesting that using SENP1 inhibitors can simultaneously enhance the SUMOylation of the two substrates. Nevertheless, while c-MYC SUMOylation promotes its degradation and thereby exerts suppressive effects on breast cancer [105], AKT SUMOylation enhances AKT activity to drive breast cancer progression [84]. Consequently, broadly targeting SUMOylation enzymes to either augment or diminish global SUMOylation might compromise therapeutic efficacy due to potential off-target effects. To effectively inhibit breast cancer growth, intervention strategies need to be more precise and targeted, focusing on specific substrates and sites.

Previous discussion mentioned the crosstalk between SUMOylation and other PTMs, including phosphorylation, ubiquitination, acetylation, and methylation in breast cancer. However, a substrate protein always contains multiple types of PTM. These PTMs coordinately regulate the function of substrate. As mentioned above, IκBα has multiple sites for phosphorylation, SUMOylation, and ubiquitination; here, phosphorylation at certain sites depresses SUMOylation, facilitating ubiquitination because the same site is shared by both modifications. Similarly, the tumor suppressor p53 also contains multiple PTMs, such as phosphorylation, SUMOylation, ubiquitination, and acetylation. SUMOylation of p53 at K386 inhibits its acetylation by p300 and decreases DNA binding activity. These results suggest the central role SUMOylation plays in the PTM network. Thus, to achieve effective therapeutic outcomes without causing unintended consequences, the inhibition of SUMOylation or the crosstalk between SUMOylation and other PTMs must be targeted specifically rather than relying on enzymes that globally promote or remove SUMOylation. To gain it, specific PDSMs and SUMOylation modification sites for a certain substrate are needed to be characterized for specifically regulation.

Further studies are warranted to elucidate the balance between SUMOylation and other PTMs in cancers, particularly under infection–inflammation-associated events. To this end, additional studies are suggested for the following: (1) performing a global RNA sequencing or microarray analysis of SUMOylation E3 ligases in different primary cancer samples and using bioinformatics tools to provide clues and predict the targets of E3 ligase; (2) analyzing cytokine profiles using microarray to characterize the physiochemical properties of the tumor microenvironment to associate with the type of SUMOylation E3 ligase that is active. The insights gained from these studies will be vital for developing improved combinatorial therapeutic strategies with a well-balanced approach to control cancer cell death without affecting the survival of normal cells. (3) SUMOylation has strong heterogeneity and cannot be generalized. It has both enhancing and inhibitory effects on breast cancer and should be considered comprehensively and precisely. The process of drug development should focus on targeting specific sequences of specific target proteins, thereby improving accuracy and specificity and decreasing side effects by affecting this interaction. (4) Methods could be developed to construct SUMO chips because of the heterogeneity of SUMOylation; these chips can be combined with mass spectrometry to comprehensively analyze the relationship between SUMOylation and other PTMs. (5) In addition to SUMOylation, the crosstalk, among other modifications, can be explored to clarify the PTM network, facilitating the better development of drugs and therapeutic targets.

In conclusion, SUMOylation plays a vital role in breast cancer development; however, it does not regulate the biological characteristics of substrate proteins in a single manner but via crosstalk with various other PTMs. The crosstalk may potentially be used in breast cancer treatment. We believe that a deep understanding of the crosstalk between SUMOylation and other PTMs may facilitate a novel treatment for breast cancer.

## Data Availability

Not applicable.

## Abbreviations

PTM: Post-translational modification
SUMO: Small ubiquitin-like modifier
ER: Estrogen receptor
PR: Progesterone receptor
HER: Human epidermal growth factor receptor
TNBC: Triple-negative breast cancer
BRCA: Breast cancer susceptibility gene
TP53: Tumor protein p53
PIK3CA: Phosphatidylinositol-4,5-Bisphosphate 3-Kinase Catalytic Subunit Alpha
PTEN: Phosphatase and tensin homolog
CCND1: Cyclin D1
FGFR1: Fibroblast growth factor receptor 1
EMT: Epithelial–mesenchymal transition
SENP: SUMO-specific protease
TGF-β: Transforming growth factor beta
KLF8: Krüppel-like factor 8
STAT: Signal transducer and activator of transcription
IFNγ: Interferon gamma
IκBα: Inhibitor of kappa B alpha
AKT: Rac-alpha serine/threonine-protein kinase
PDSM: Phosphorylation-dependent SUMOylation motif
HSF1: Heat shock transcription factor 1
MEF2: Myocyte enhancer factor 2
PPARγ: Peroxisome proliferator activated receptor gamma
NCoR: Nuclear receptor corepressor
NDSM: Negative charge-dependent SUMOylation motif
ELK1: ETS domain-containing protein Elk1
PML: Promyelocytic leukemia
MZF1: Myeloid zinc finger-1
FOXM1: Forkhead box M1
RNF: Ring finger protein
HERC2: HECT and RLD domain containing E3 ubiquitin protein ligase 2
STUbL: SUMO-targeted E3 ubiquitin ligase
SIM: SUMO-interacting motif
BCA: Breast cancer-associated gene
DLf: Delta-lactoferrin
PRB: Progesterone receptor isoform B
ZFHX3: Zinc finger homeobox 3
PES1: Pescadillo ribosomal biogenesis factor 1
HDAC: Histone deacetylase
PIASy: Protein inhibitor of activated STAT 4
TRIM24: Tripartite motif-containing protein 24
AIB1: Breast cancer 1
HOXB13: Homeobox B13
KAP1: KRAB domain-associated protein 1
HIC1: Hypermethylated in cancer 1
H4K20me1: Monomethylates lysine 20 of histone H4
SAE: SUMO1 Activating Enzyme Subunit 1
FIS1: Fission protein 1
NEDD8: Neural precursor cell-expressed developmentally downregulated 8
MDM2: Murine double minute 2
RPL11: Ribosomal protein L11
EGF: Epidermal growth factor
PI3K: Phosphatidylinositol 3-kinase
CD: Cluster of differentiation

## References

[1] Siegel RL, Miller KD, Jemal A. Cancer statistics, 2020. CA Cancer J Clin. 2020;70:7–30. 31912902 10.3322/caac.21590

[2] Bray F, Ferlay J, Soerjomataram I, Siegel RL, Torre LA, Jemal A. Global cancer statistics 2018: GLOBOCAN estimates of incidence and mortality worldwide for 36 cancers in 185 countries. CA Cancer J Clin. 2018;68:394–424. 30207593 10.3322/caac.21492

[3] Bray F, Ferlay J, Laversanne M, Brewster DH, Gombe Mbalawa C, Kohler B, et al. Cancer Incidence in Five Continents: Inclusion criteria, highlights from Volume X and the global status of cancer registration. Int J Cancer. 2015;137:2060–71. 26135522 10.1002/ijc.29670

[4] Harbeck N, Penault-Llorca F, Cortes J, Gnant M, Houssami N, Poortmans P, et al. Breast cancer. Nat Rev Dis Primers. 2019;5:66. 31548545 10.1038/s41572-019-0111-2

[5] Chen W, Zheng R, Baade PD, Zhang S, Zeng H, Bray F, et al. Cancer statistics in China, 2015. CA Cancer J Clin. 2016;66:115–32. 26808342 10.3322/caac.21338

[6] Siegel RL, Miller KD, Jemal A. Cancer statistics, 2019. CA Cancer J Clin. 2019;69:7–34. 30620402 10.3322/caac.21551

[7] Medina MA, Oza G, Sharma A, Arriaga LG, Hernandez Hernandez JM, Rotello VM, et al. Triple-negative breast cancer: a review of conventional and advanced therapeutic strategies. Int J Environ Res Public Health. 2020. 10.3390/ijerph17062078. 10.3390/ijerph17062078 PMC7143295 32245065

[8] Hammond ME, Hayes DF, Dowsett M, Allred DC, Hagerty KL, Badve S, et al. American Society of Clinical Oncology/College Of American Pathologists guideline recommendations for immunohistochemical testing of estrogen and progesterone receptors in breast cancer. J Clin Oncol. 2010;28:2784–95. 20404251 10.1200/JCO.2009.25.6529 PMC2881855

[9] Loibl S, Poortmans P, Morrow M, Denkert C, Curigliano G. Breast cancer. Lancet. 2021;397:1750–69. 33812473 10.1016/S0140-6736(20)32381-3

[10] Inic Z, Zegarac M, Inic M, Markovic I, Kozomara Z, Djurisic I, et al. Difference between Luminal A and Luminal B Subtypes According to Ki-67, Tumor Size, and Progesterone Receptor Negativity Providing Prognostic Information. Clin Med Insights Oncol. 2014;8:107–11. 25249766 10.4137/CMO.S18006 PMC4167319

[11] Viale G, Hanlon Newell AE, Walker E, Harlow G, Bai I, Russo L, et al. Ki-67 (30–9) scoring and differentiation of Luminal A- and Luminal B-like breast cancer subtypes. Breast Cancer Res Treat. 2019;178:451–8. 31422497 10.1007/s10549-019-05402-w PMC6797656

[12] Lehmann BD, Bauer JA, Chen X, Sanders ME, Chakravarthy AB, Shyr Y, et al. Identification of human triple-negative breast cancer subtypes and preclinical models for selection of targeted therapies. J Clin Invest. 2011;121:2750–67. 21633166 10.1172/JCI45014 PMC3127435

[13] Ren JX, Gong Y, Ling H, Hu X, Shao ZM. Racial/ethnic differences in the outcomes of patients with metastatic breast cancer: contributions of demographic, socioeconomic, tumor and metastatic characteristics. Breast Cancer Res Treat. 2019;173:225–37. 30293212 10.1007/s10549-018-4956-y PMC6394580

[14] Kuchenbaecker KB, Hopper JL, Barnes DR, Phillips KA, Mooij TM, Roos-Blom MJ, et al. Risks of breast, ovarian, and contralateral breast cancer for BRCA1 and BRCA2 mutation carriers. JAMA. 2017;317:2402–16. 28632866 10.1001/jama.2017.7112

[15] Chen S, Parmigiani G. Meta-analysis of BRCA1 and BRCA2 penetrance. J Clin Oncol. 2007;25:1329–33. 17416853 10.1200/JCO.2006.09.1066 PMC2267287

[16] Huen MS, Sy SM, Chen J. BRCA1 and its toolbox for the maintenance of genome integrity. Nat Rev Mol Cell Biol. 2010;11:138–48. 20029420 10.1038/nrm2831 PMC3899800

[17] Pohl-Rescigno E, Hauke J, Loibl S, Mobus V, Denkert C, Fasching PA, et al. Association of germline variant status with therapy response in high-risk early-stage breast cancer: a secondary analysis of the GeparOcto randomized clinical trial. JAMA Oncol. 2020;6:744–8. 32163106 10.1001/jamaoncol.2020.0007 PMC7068666

[18] Nik-Zainal S, Davies H, Staaf J, Ramakrishna M, Glodzik D, Zou X, et al. Landscape of somatic mutations in 560 breast cancer whole-genome sequences. Nature. 2016;534:47–54. 27135926 10.1038/nature17676 PMC4910866

[19] Tsang JYS, Tse GM. Molecular classification of breast cancer. Adv Anat Pathol. 2020;27:27–35. 31045583 10.1097/PAP.0000000000000232

[20] Liu J, Wang Q, Kang Y, Xu S, Pang D. Unconventional protein post-translational modifications: the helmsmen in breast cancer. Cell Biosci. 2022;12:22. 35216622 10.1186/s13578-022-00756-z PMC8881842

[21] Vasilescu J, Smith JC, Ethier M, Figeys D. Proteomic analysis of ubiquitinated proteins from human MCF-7 breast cancer cells by immunoaffinity purification and mass spectrometry. J Proteome Res. 2005;4:2192–200. 16335966 10.1021/pr050265i

[22] Luo M. Chemical and biochemical perspectives of protein lysine methylation. Chem Rev. 2018;118:6656–705. 29927582 10.1021/acs.chemrev.8b00008 PMC6668730

[23] Hendriks IA, Vertegaal AC. A comprehensive compilation of SUMO proteomics. Nat Rev Mol Cell Biol. 2016;17:581–95. 27435506 10.1038/nrm.2016.81

[24] Heo KS. Regulation of post-translational modification in breast cancer treatment. BMB Rep. 2019;52:113–8. 30638182 10.5483/BMBRep.2019.52.2.017 PMC6443327

[25] Geng P, Zhang Y, Liu X, Zhang N, Liu Y, Liu X, et al. Automethylation of protein arginine methyltransferase 7 and its impact on breast cancer progression. FASEB J. 2017;31:2287–300. 28188177 10.1096/fj.201601196R

[26] Li H, Guan Y. Machine learning empowers phosphoproteome prediction in cancers. Bioinformatics. 2020;36:859–64. 31410451 10.1093/bioinformatics/btz639 PMC7868059

[27] Guo P, Chen W, Li H, Li M, Li L. The histone acetylation modifications of breast cancer and their therapeutic implications. Pathol Oncol Res. 2018;24:807–13. 29948617 10.1007/s12253-018-0433-5

[28] Kharman-Biz A, Gao H, Ghiasvand R, Haldosen LA, Zendehdel K. Expression of the three components of linear ubiquitin assembly complex in breast cancer. PLoS ONE. 2018;13: e0197183. 29763465 10.1371/journal.pone.0197183 PMC5953448

[29] Pal A, Donato NJ. Ubiquitin-specific proteases as therapeutic targets for the treatment of breast cancer. Breast Cancer Res. 2014;16:461. 25606592 10.1186/s13058-014-0461-3 PMC4384352

[30] Stadler SC, Vincent CT, Fedorov VD, Patsialou A, Cherrington BD, Wakshlag JJ, et al. Dysregulation of PAD4-mediated citrullination of nuclear GSK3beta activates TGF-beta signaling and induces epithelial-to-mesenchymal transition in breast cancer cells. Proc Natl Acad Sci U S A. 2013;110:11851–6. 23818587 10.1073/pnas.1308362110 PMC3718105

[31] Yao R, Wang Y, Han D, Ma Y, Ma M, Zhao Y, et al. Lysines 207 and 325 methylation of WDR5 catalyzed by SETD6 promotes breast cancer cell proliferation and migration. Oncol Rep. 2018;40:3069–77. 30226578 10.3892/or.2018.6669

[32] Scott DA, Drake RR. Glycosylation and its implications in breast cancer. Expert Rev Proteomics. 2019;16:665–80. 31314995 10.1080/14789450.2019.1645604 PMC6702063

[33] Liu HY, Liu YY, Yang F, Zhang L, Zhang FL, Hu X, et al. Acetylation of MORC2 by NAT10 regulates cell-cycle checkpoint control and resistance to DNA-damaging chemotherapy and radiotherapy in breast cancer. Nucleic Acids Res. 2020;48:3638–56. 32112098 10.1093/nar/gkaa130 PMC7144926

[34] Anderson AM, Ragan MA. Palmitoylation: a protein S-acylation with implications for breast cancer. NPJ Breast Cancer. 2016;2:16028. 28721385 10.1038/npjbcancer.2016.28 PMC5515344

[35] Qin Y, Yuan H, Chen X, Yang X, Xing Z, Shen Y, et al. SUMOylation wrestles with the occurrence and development of breast cancer. Front Oncol. 2021;11: 659661. 33968766 10.3389/fonc.2021.659661 PMC8097099

[36] Rabellino A, Khanna KK. The implication of the SUMOylation pathway in breast cancer pathogenesis and treatment. Crit Rev Biochem Mol Biol. 2020;55:54–70. 32183544 10.1080/10409238.2020.1738332

[37] Kamada S, Takeiwa T, Ikeda K, Horie K, Inoue S. Emerging roles of COX7RP and mitochondrial oxidative phosphorylation in breast cancer. Front Cell Dev Biol. 2022;10: 717881. 35178385 10.3389/fcell.2022.717881 PMC8844363

[38] Kastrati I, Semina S, Gordon B, Smart E. Insights into how phosphorylation of estrogen receptor at serine 305 modulates tamoxifen activity in breast cancer. Mol Cell Endocrinol. 2019;483:97–101. 30659843 10.1016/j.mce.2019.01.014 PMC6368394

[39] Naik SK, Lam EW, Parija M, Prakash S, Jiramongkol Y, Adhya AK, et al. NEDDylation negatively regulates ERRbeta expression to promote breast cancer tumorigenesis and progression. Cell Death Dis. 2020;11:703. 32839427 10.1038/s41419-020-02838-7 PMC7445179

[40] Mu R, Ma Z, Lu C, Wang H, Cheng X, Tuo B, et al. Role of succinylation modification in thyroid cancer and breast cancer. Am J Cancer Res. 2021;11:4683–99. 34765287 PMC8569371

[41] Mishra D, Patel V, Banerjee D. Nitric oxide and S-nitrosylation in cancers: emphasis on breast cancer. Breast Cancer (Auckl). 2020;14:1178223419882688. 32030066 10.1177/1178223419882688 PMC6977095

[42] Mirecka A, Morawiec Z, Wozniak K. Genetic polymorphism of SUMO-specific cysteine proteases—SENP1 and SENP2 in breast cancer. Pathol Oncol Res. 2016;22:817–23. 27178176 10.1007/s12253-016-0064-7 PMC5031717

[43] Hershko A, Ciechanover A. The ubiquitin system. Annu Rev Biochem. 1998;67:425–79. 9759494 10.1146/annurev.biochem.67.1.425

[44] Seeler JS, Dejean A. SUMO and the robustness of cancer. Nat Rev Cancer. 2017;17:184–97. 28134258 10.1038/nrc.2016.143

[45] Horibata S, Rogers KE, Sadegh D, Anguish LJ, McElwee JL, Shah P, et al. Role of peptidylarginine deiminase 2 (PAD2) in mammary carcinoma cell migration. BMC Cancer. 2017;17:378. 28549415 10.1186/s12885-017-3354-x PMC5446677

[46] Cho Y, Kang HG, Kim SJ, Lee S, Jee S, Ahn SG, et al. Post-translational modification of OCT4 in breast cancer tumorigenesis. Cell Death Differ. 2018;25:1781–95. 29511337 10.1038/s41418-018-0079-6 PMC6180041

[47] Shi J, Wang Y, Zeng L, Wu Y, Deng J, Zhang Q, et al. Disrupting the interaction of BRD4 with diacetylated Twist suppresses tumorigenesis in basal-like breast cancer. Cancer Cell. 2014;25:210–25. 24525235 10.1016/j.ccr.2014.01.028 PMC4004960

[48] Jiang K, Gao Y, Hou W, Tian F, Ying W, Li L, et al. Proteomic analysis of O-GlcNAcylated proteins in invasive ductal breast carcinomas with and without lymph node metastasis. Amino Acids. 2016;48:365–74. 26374642 10.1007/s00726-015-2089-8

[49] Matunis MJ, Coutavas E, Blobel G. A novel ubiquitin-like modification modulates the partitioning of the Ran-GTPase-activating protein RanGAP1 between the cytosol and the nuclear pore complex. J Cell Biol. 1996;135:1457–70. 8978815 10.1083/jcb.135.6.1457 PMC2133973

[50] Mahajan R, Delphin C, Guan T, Gerace L, Melchior F. A small ubiquitin-related polypeptide involved in targeting RanGAP1 to nuclear pore complex protein RanBP2. Cell. 1997;88:97–107. 9019411 10.1016/S0092-8674(00)81862-0

[51] Pichler A, Fatouros C, Lee H, Eisenhardt N. SUMO conjugation—a mechanistic view. Biomol Concepts. 2017;8:13–36. 28284030 10.1515/bmc-2016-0030

[52] Saitoh H, Hinchey J. Functional heterogeneity of small ubiquitin-related protein modifiers SUMO-1 versus SUMO-2/3. J Biol Chem. 2000;275:6252–8. 10692421 10.1074/jbc.275.9.6252

[53] Tatham MH, Jaffray E, Vaughan OA, Desterro JM, Botting CH, Naismith JH, et al. Polymeric chains of SUMO-2 and SUMO-3 are conjugated to protein substrates by SAE1/SAE2 and Ubc9. J Biol Chem. 2001;276:35368–74. 11451954 10.1074/jbc.M104214200

[54] Matic I, van Hagen M, Schimmel J, Macek B, Ogg SC, Tatham MH, et al. In vivo identification of human small ubiquitin-like modifier polymerization sites by high accuracy mass spectrometry and an in vitro to in vivo strategy. Mol Cell Proteomics. 2008;7:132–44. 17938407 10.1074/mcp.M700173-MCP200 PMC3840926

[55] Woo CH, Abe J. SUMO–a post-translational modification with therapeutic potential? Curr Opin Pharmacol. 2010;10:146–55. 20079693 10.1016/j.coph.2009.12.001 PMC3000872

[56] Yeh ET. SUMOylation and De-SUMOylation: wrestling with life’s processes. J Biol Chem. 2009;284:8223–7. 19008217 10.1074/jbc.R800050200 PMC2659178

[57] Gong L, Millas S, Maul GG, Yeh ET. Differential regulation of sentrinized proteins by a novel sentrin-specific protease. J Biol Chem. 2000;275:3355–9. 10652325 10.1074/jbc.275.5.3355

[58] Chang HM, Yeh ETH. SUMO: from bench to bedside. Physiol Rev. 2020;100:1599–619. 32666886 10.1152/physrev.00025.2019 PMC7717128

[59] Best JL, Ganiatsas S, Agarwal S, Changou A, Salomoni P, Shirihai O, et al. SUMO-1 protease-1 regulates gene transcription through PML. Mol Cell. 2002;10:843–55. 12419228 10.1016/S1097-2765(02)00699-8

[60] Kadoya T, Yamamoto H, Suzuki T, Yukita A, Fukui A, Michiue T, et al. Desumoylation activity of Axam, a novel Axin-binding protein, is involved in downregulation of beta-catenin. Mol Cell Biol. 2002;22:3803–19. 11997515 10.1128/MCB.22.11.3803-3819.2002 PMC133821

[61] Zhang H, Saitoh H, Matunis MJ. Enzymes of the SUMO modification pathway localize to filaments of the nuclear pore complex. Mol Cell Biol. 2002;22:6498–508. 12192048 10.1128/MCB.22.18.6498-6508.2002 PMC135644

[62] Qiu C, Wang Y, Zhao H, Qin L, Shi Y, Zhu X, et al. The critical role of SENP1-mediated GATA2 deSUMOylation in promoting endothelial activation in graft arteriosclerosis. Nat Commun. 2017;8:15426. 28569748 10.1038/ncomms15426 PMC5461500

[63] Zhu X, Qiu C, Wang Y, Jiang Y, Chen Y, Fan L, et al. FGFR1 SUMOylation coordinates endothelial angiogenic signaling in angiogenesis. Proc Natl Acad Sci U S A. 2022;119: e2202631119. 35733256 10.1073/pnas.2202631119 PMC9245619

[64] Zhu X, Ding S, Qiu C, Shi Y, Song L, Wang Y, et al. SUMOylation negatively regulates angiogenesis by targeting endothelial NOTCH signaling. Circ Res. 2017;121:636–49. 28760777 10.1161/CIRCRESAHA.117.310696 PMC5581236

[65] Zhou X, Jiang Y, Wang Y, Fan L, Zhu Y, Chen Y, et al. Endothelial FIS1 DeSUMOylation protects against hypoxic pulmonary hypertension. Circ Res. 2023;133:508–31. 37589160 10.1161/CIRCRESAHA.122.321200

[66] Ren R, Ding S, Ma K, Jiang Y, Wang Y, Chen J, et al. SUMOylation fine-tunes endothelial HEY1 in the regulation of angiogenesis. Circ Res. 2024;134:203–22. 38166414 10.1161/CIRCRESAHA.123.323398 PMC10872267

[67] Vialter A, Vincent A, Demidem A, Morvan D, Stepien G, Venezia ND, et al. Cell cycle-dependent conjugation of endogenous BRCA1 protein with SUMO-2/3. Biochim Biophys Acta. 2011;1810:432–8. 21147198 10.1016/j.bbagen.2010.12.001

[68] Huang Z, Barker D, Gibbins JM, Dash PR. Talin is a substrate for SUMOylation in migrating cancer cells. Exp Cell Res. 2018;370:417–25. 30003879 10.1016/j.yexcr.2018.07.005 PMC6117455

[69] Kang JS, Saunier EF, Akhurst RJ, Derynck R. The type I TGF-beta receptor is covalently modified and regulated by sumoylation. Nat Cell Biol. 2008;10:654–64. 18469808 10.1038/ncb1728 PMC2649123

[70] Chang CC, Lin DY, Fang HI, Chen RH, Shih HM. Daxx mediates the small ubiquitin-like modifier-dependent transcriptional repression of Smad4. J Biol Chem. 2005;280:10164–73. 15637079 10.1074/jbc.M409161200

[71] Chen CH, Chang CC, Lee TH, Luo M, Huang P, Liao PH, et al. SENP1 deSUMOylates and regulates Pin1 protein activity and cellular function. Cancer Res. 2013;73:3951–62. 23633483 10.1158/0008-5472.CAN-12-4360 PMC3818121

[72] Cohen P. The origins of protein phosphorylation. Nat Cell Biol. 2002;4:E127-130. 11988757 10.1038/ncb0502-e127

[73] Burnett G, Kennedy EP. The enzymatic phosphorylation of proteins. J Biol Chem. 1954;211:969–80. 13221602 10.1016/S0021-9258(18)71184-8

[74] Fischer EH, Krebs EG. Conversion of phosphorylase b to phosphorylase a in muscle extracts. J Biol Chem. 1955;216:121–32. 13252012 10.1016/S0021-9258(19)52289-X

[75] Vertegaal ACO. Signalling mechanisms and cellular functions of SUMO. Nat Rev Mol Cell Biol. 2022;23:715–31. 35750927 10.1038/s41580-022-00500-y

[76] Lu H, Hu L, Li T, Lahiri S, Shen C, Wason MS, et al. A novel role of Kruppel-like factor 8 in DNA repair in breast cancer cells. J Biol Chem. 2012;287:43720–9. 23105099 10.1074/jbc.M112.418053 PMC3527957

[77] Hietakangas V, Anckar J, Blomster HA, Fujimoto M, Palvimo JJ, Nakai A, et al. PDSM, a motif for phosphorylation-dependent SUMO modification. Proc Natl Acad Sci U S A. 2006;103:45–50. 16371476 10.1073/pnas.0503698102 PMC1324973

[78] Yu L, Ji W, Zhang H, Renda MJ, He Y, Lin S, et al. SENP1-mediated GATA1 deSUMOylation is critical for definitive erythropoiesis. J Exp Med. 2010;207:1183–95. 20457756 10.1084/jem.20092215 PMC2882842

[79] Feng L, Li W, Li X, Li X, Ran Y, Yang X, et al. N-MYC-interacting protein enhances type II interferon signaling by inhibiting STAT1 sumoylation. FASEB J. 2023;37: e23281. 37933920 10.1096/fj.202301450RR

[80] Vu EH, Kraus RJ, Mertz JE. Phosphorylation-dependent sumoylation of estrogen-related receptor alpha1. Biochemistry. 2007;46:9795–804. 17676930 10.1021/bi700316g

[81] Picard N, Caron V, Bilodeau S, Sanchez M, Mascle X, Aubry M, et al. Identification of estrogen receptor beta as a SUMO-1 target reveals a novel phosphorylated sumoylation motif and regulation by glycogen synthase kinase 3beta. Mol Cell Biol. 2012;32:2709–21. 22586270 10.1128/MCB.06624-11 PMC3416183

[82] Liu Y, Tavana O, Gu W. p53 modifications: exquisite decorations of the powerful guardian. J Mol Cell Biol. 2019;11:564–77. 31282934 10.1093/jmcb/mjz060 PMC6736412

[83] Wang X, Peng H, Huang Y, Kong W, Cui Q, Du J, et al. Post-translational modifications of ikappabalpha: the state of the art. Front Cell Dev Biol. 2020;8: 574706. 33224945 10.3389/fcell.2020.574706 PMC7674170

[84] de la Cruz-Herrera CF, Campagna M, Lang V, del Carmen G-S, Marcos-Villar L, Rodriguez MS, et al. SUMOylation regulates AKT1 activity. Oncogene. 2015;34:1442–50. 24704831 10.1038/onc.2014.48

[85] Xiao M, Bian Q, Lao Y, Yi J, Sun X, Sun X, et al. SENP3 loss promotes M2 macrophage polarization and breast cancer progression. Mol Oncol. 2022;16:1026–44. 33932085 10.1002/1878-0261.12967 PMC8847990

[86] Li Y, Xing Y, Wang X, Hu B, Zhao X, Zhang H, et al. PAK5 promotes RNA helicase DDX5 sumoylation and miRNA-10b processing in a kinase-dependent manner in breast cancer. Cell Rep. 2021;37: 110127. 34936874 10.1016/j.celrep.2021.110127

[87] Abdel-Hafiz HA, Horwitz KB. Post-translational modifications of the progesterone receptors. J Steroid Biochem Mol Biol. 2014;140:80–9. 24333793 10.1016/j.jsbmb.2013.12.008 PMC3923415

[88] Mohideen F, Capili AD, Bilimoria PM, Yamada T, Bonni A, Lima CD. A molecular basis for phosphorylation-dependent SUMO conjugation by the E2 UBC9. Nat Struct Mol Biol. 2009;16:945–52. 19684601 10.1038/nsmb.1648 PMC2771680

[89] Schilling G. RADIO ASTRONOMY. Fast radio bursts tease astronomers. Science. 2016;351:1012–3. 26941294 10.1126/science.351.6277.1012

[90] Yang SH, Galanis A, Witty J, Sharrocks AD. An extended consensus motif enhances the specificity of substrate modification by SUMO. EMBO J. 2006;25:5083–93. 17036045 10.1038/sj.emboj.7601383 PMC1630412

[91] Lin CH, Liu SY, Lee EH. SUMO modification of Akt regulates global SUMOylation and substrate SUMOylation specificity through Akt phosphorylation of Ubc9 and SUMO1. Oncogene. 2016;35:595–607. 25867063 10.1038/onc.2015.115

[92] Su X, Mancuso DJ, Bickel PE, Jenkins CM, Gross RW. Small interfering RNA knockdown of calcium-independent phospholipases A2 beta or gamma inhibits the hormone-induced differentiation of 3T3-L1 preadipocytes. J Biol Chem. 2004;279:21740–8. 15024020 10.1074/jbc.M314166200

[93] Muller S, Matunis MJ, Dejean A. Conjugation with the ubiquitin-related modifier SUMO-1 regulates the partitioning of PML within the nucleus. EMBO J. 1998;17:61–70. 9427741 10.1093/emboj/17.1.61 PMC1170358

[94] Muller S, Berger M, Lehembre F, Seeler JS, Haupt Y, Dejean A. c-Jun and p53 activity is modulated by SUMO-1 modification. J Biol Chem. 2000;275:13321–9. 10788439 10.1074/jbc.275.18.13321

[95] Yang SH, Jaffray E, Hay RT, Sharrocks AD. Dynamic interplay of the SUMO and ERK pathways in regulating Elk-1 transcriptional activity. Mol Cell. 2003;12:63–74. 12887893 10.1016/S1097-2765(03)00265-X

[96] Yang SH, Jaffray E, Senthinathan B, Hay RT, Sharrocks AD. SUMO and transcriptional repression: dynamic interactions between the MAP kinase and SUMO pathways. Cell Cycle. 2003;2:528–30. 14504467 10.4161/cc.2.6.597

[97] Brix DM, Tvingsholm SA, Hansen MB, Clemmensen KB, Ohman T, Siino V, et al. Release of transcriptional repression via ErbB2-induced, SUMO-directed phosphorylation of myeloid zinc finger-1 serine 27 activates lysosome redistribution and invasion. Oncogene. 2019;38:3170–84. 30622337 10.1038/s41388-018-0653-x PMC6525100

[98] Kongsema M, Zona S, Karunarathna U, Cabrera E, Man EP, Yao S, et al. RNF168 cooperates with RNF8 to mediate FOXM1 ubiquitination and degradation in breast cancer epirubicin treatment. Oncogenesis. 2016;5: e252. 27526106 10.1038/oncsis.2016.57 PMC5007831

[99] Myatt SS, Kongsema M, Man CW, Kelly DJ, Gomes AR, Khongkow P, et al. SUMOylation inhibits FOXM1 activity and delays mitotic transition. Oncogene. 2014;33:4316–29. 24362530 10.1038/onc.2013.546 PMC4096495

[100] Myatt SS, Lam EW. Targeting FOXM1. Nat Rev Cancer. 2008;8:242. 18297052 10.1038/nrc2223-c2

[101] Kwok JM, Myatt SS, Marson CM, Coombes RC, Constantinidou D, Lam EW. Thiostrepton selectively targets breast cancer cells through inhibition of forkhead box M1 expression. Mol Cancer Ther. 2008;7:2022–32. 18645012 10.1158/1535-7163.MCT-08-0188

[102] Kwok JM, Peck B, Monteiro LJ, Schwenen HD, Millour J, Coombes RC, et al. FOXM1 confers acquired cisplatin resistance in breast cancer cells. Mol Cancer Res. 2010;8:24–34. 20068070 10.1158/1541-7786.MCR-09-0432 PMC2809047

[103] McGovern UB, Francis RE, Peck B, Guest SK, Wang J, Myatt SS, et al. Gefitinib (Iressa) represses FOXM1 expression via FOXO3a in breast cancer. Mol Cancer Ther. 2009;8:582–91. 19276163 10.1158/1535-7163.MCT-08-0805

[104] Khongkow P, Gomes AR, Gong C, Man EP, Tsang JW, Zhao F, et al. Paclitaxel targets FOXM1 to regulate KIF20A in mitotic catastrophe and breast cancer paclitaxel resistance. Oncogene. 2016;35:990–1002. 25961928 10.1038/onc.2015.152 PMC4538879

[105] Gonzalez-Prieto R, Cuijpers SA, Kumar R, Hendriks IA, Vertegaal AC. c-Myc is targeted to the proteasome for degradation in a SUMOylation-dependent manner, regulated by PIAS1, SENP7 and RNF4. Cell Cycle. 2015;14:1859–72. 25895136 10.1080/15384101.2015.1040965 PMC4613540

[106] Kalkat M, Chan PK, Wasylishen AR, Srikumar T, Kim SS, Ponzielli R, et al. Identification of c-MYC SUMOylation by mass spectrometry. PLoS ONE. 2014;9: e115337. 25522242 10.1371/journal.pone.0115337 PMC4270761

[107] Sun XX, Chen Y, Su Y, Wang X, Chauhan KM, Liang J, et al. SUMO protease SENP1 deSUMOylates and stabilizes c-Myc. Proc Natl Acad Sci U S A. 2018;115:10983–8. 30305424 10.1073/pnas.1802932115 PMC6205424

[108] Lamoliatte F, McManus FP, Maarifi G, Chelbi-Alix MK, Thibault P. Uncovering the SUMOylation and ubiquitylation crosstalk in human cells using sequential peptide immunopurification. Nat Commun. 2017;8:14109. 28098164 10.1038/ncomms14109 PMC5253644

[109] Chen Y, Sun XX, Sears RC, Dai MS. Writing and erasing MYC ubiquitination and SUMOylation. Genes Dis. 2019;6:359–71. 31832515 10.1016/j.gendis.2019.05.006 PMC6889025

[110] Gareau JR, Lima CD. The SUMO pathway: emerging mechanisms that shape specificity, conjugation and recognition. Nat Rev Mol Cell Biol. 2010;11:861–71. 21102611 10.1038/nrm3011 PMC3079294

[111] Moldovan GL, Pfander B, Jentsch S. PCNA controls establishment of sister chromatid cohesion during S phase. Mol Cell. 2006;23:723–32. 16934511 10.1016/j.molcel.2006.07.007

[112] Desterro JM, Rodriguez MS, Hay RT. SUMO-1 modification of IkappaBalpha inhibits NF-kappaB activation. Mol Cell. 1998;2:233–9. 9734360 10.1016/S1097-2765(00)80133-1

[113] Zhao Y, Brickner JR, Majid MC, Mosammaparast N. Crosstalk between ubiquitin and other post-translational modifications on chromatin during double-strand break repair. Trends Cell Biol. 2014;24:426–34. 24569222 10.1016/j.tcb.2014.01.005 PMC4074573

[114] Xu Y, Plechanovova A, Simpson P, Marchant J, Leidecker O, Kraatz S, et al. Structural insight into SUMO chain recognition and manipulation by the ubiquitin ligase RNF4. Nat Commun. 2014;5:4217. 24969970 10.1038/ncomms5217 PMC4083429

[115] Kumar R, Sabapathy K. RNF4-A Paradigm for SUMOylation-Mediated Ubiquitination. Proteomics. 2019;19: e1900185. 31566917 10.1002/pmic.201900185

[116] Guzzo CM, Berndsen CE, Zhu J, Gupta V, Datta A, Greenberg RA, et al. RNF4-dependent hybrid SUMO-ubiquitin chains are signals for RAP80 and thereby mediate the recruitment of BRCA1 to sites of DNA damage. Sci Signal. 2012;5:ra88. 23211528 10.1126/scisignal.2003485 PMC4131685

[117] Galanty Y, Belotserkovskaya R, Coates J, Jackson SP. RNF4, a SUMO-targeted ubiquitin E3 ligase, promotes DNA double-strand break repair. Genes Dev. 2012;26:1179–95. 22661229 10.1101/gad.188284.112 PMC3371407

[118] Yin Y, Seifert A, Chua JS, Maure JF, Golebiowski F, Hay RT. SUMO-targeted ubiquitin E3 ligase RNF4 is required for the response of human cells to DNA damage. Genes Dev. 2012;26:1196–208. 22661230 10.1101/gad.189274.112 PMC3371408

[119] Rojas-Fernandez A, Plechanovova A, Hattersley N, Jaffray E, Tatham MH, Hay RT. SUMO chain-induced dimerization activates RNF4. Mol Cell. 2014;53:880–92. 24656128 10.1016/j.molcel.2014.02.031 PMC3991395

[120] Lallemand-Breitenbach V, Jeanne M, Benhenda S, Nasr R, Lei M, Peres L, et al. Arsenic degrades PML or PML-RARalpha through a SUMO-triggered RNF4/ubiquitin-mediated pathway. Nat Cell Biol. 2008;10:547–55. 18408733 10.1038/ncb1717

[121] Tatham MH, Geoffroy MC, Shen L, Plechanovova A, Hattersley N, Jaffray EG, et al. RNF4 is a poly-SUMO-specific E3 ubiquitin ligase required for arsenic-induced PML degradation. Nat Cell Biol. 2008;10:538–46. 18408734 10.1038/ncb1716

[122] Shi Y, Castro-Gonzalez S, Chen Y, Serra-Moreno R. Effects of the SUMO ligase BCA2 on metabolic activity, cell proliferation, cell migration, cell cycle, and the regulation of NF-kappaB and IRF1 in different breast epithelial cellular contexts. Front Cell Dev Biol. 2021;9: 711481. 34589482 10.3389/fcell.2021.711481 PMC8473798

[123] Escobar-Ramirez A, Vercoutter-Edouart AS, Mortuaire M, Huvent I, Hardiville S, Hoedt E, et al. Modification by SUMOylation controls both the transcriptional activity and the stability of delta-lactoferrin. PLoS ONE. 2015;10: e0129965. 26090800 10.1371/journal.pone.0129965 PMC4474976

[124] Zhang PJ, Zhao J, Li HY, Man JH, He K, Zhou T, et al. CUE domain containing 2 regulates degradation of progesterone receptor by ubiquitin-proteasome. EMBO J. 2007;26:1831–42. 17347654 10.1038/sj.emboj.7601602 PMC1847652

[125] Wu R, Fang J, Liu M, A J, Liu J, Chen W, et al. SUMOylation of the transcription factor ZFHX3 at Lys-2806 requires SAE1, UBC9, and PIAS2 and enhances its stability and function in cell proliferation. J Biol Chem. 2020;295:6741–6753. 10.1074/jbc.RA119.012338 PMC7212658 32249212

[126] Li S, Wang M, Qu X, Xu Z, Yang Y, Su Q, et al. SUMOylation of PES1 upregulates its stability and function via inhibiting its ubiquitination. Oncotarget. 2016;7:50522–34. 27409667 10.18632/oncotarget.10494 PMC5226600

[127] Citro S, Jaffray E, Hay RT, Seiser C, Chiocca S. A role for paralog-specific sumoylation in histone deacetylase 1 stability. J Mol Cell Biol. 2013;5:416–27. 24068740 10.1093/jmcb/mjt032

[128] Lian B, Chen X, Shen K. Inhibition of histone deacetylases attenuates tumor progression and improves immunotherapy in breast cancer. Front Immunol. 2023;14:1164514. 36969235 10.3389/fimmu.2023.1164514 PMC10034161

[129] Appikonda S, Thakkar KN, Shah PK, Dent SYR, Andersen JN, Barton MC. Cross-talk between chromatin acetylation and SUMOylation of tripartite motif-containing protein 24 (TRIM24) impacts cell adhesion. J Biol Chem. 2018;293:7476–85. 29523690 10.1074/jbc.RA118.002233 PMC5950014

[130] Sheban D, Shani T, Maor R, Aguilera-Castrejon A, Mor N, Oldak B, et al. SUMOylation of linker histone H1 drives chromatin condensation and restriction of embryonic cell fate identity. Mol Cell. 2022;82(106–122): e109. 10.1016/j.molcel.2021.11.011 PMC11822872 34875212

[131] Leonen CJA, Shimada M, Weller CE, Nakadai T, Hsu PL, Tyson EL, et al. Sumoylation of the human histone H4 tail inhibits p300-mediated transcription by RNA polymerase II in cellular extracts. Elife. 2021;10. 10.7554/eLife.67952 PMC8626089 34747692

[132] Ryu HY, Zhao D, Li J, Su D, Hochstrasser M. Histone sumoylation promotes Set3 histone-deacetylase complex-mediated transcriptional regulation. Nucleic Acids Res. 2020;48:12151–68. 33231641 10.1093/nar/gkaa1093 PMC7708062

[133] Wu SY, Chiang CM. Crosstalk between sumoylation and acetylation regulates p53-dependent chromatin transcription and DNA binding. EMBO J. 2009;28:1246–59. 19339993 10.1038/emboj.2009.83 PMC2683057

[134] Li Y, Li S, Shi X, Xin Z, Yang Y, Zhao B, et al. KLF12 promotes the proliferation of breast cancer cells by reducing the transcription of p21 in a p53-dependent and p53-independent manner. Cell Death Dis. 2023;14:313. 37156774 10.1038/s41419-023-05824-x PMC10167366

[135] Cao W, Shen R, Richard S, Liu Y, Jalalirad M, Cleary MP, et al. Inhibition of triple‑negative breast cancer proliferation and motility by reactivating p53 and inhibiting overactivated Akt. Oncol Rep. 2022;47. 10.3892/or.2021.8252 PMC8759100 34958116

[136] Li Q, Hao Q, Cao W, Li J, Wu K, Elshimali Y, et al. PP2Cdelta inhibits p300-mediated p53 acetylation via ATM/BRCA1 pathway to impede DNA damage response in breast cancer. Sci Adv. 2019;5:eaaw8417. 31663018 10.1126/sciadv.aaw8417 PMC6795508

[137] Hardiville S, Escobar-Ramirez A, Pina-Canceco S, Elass E, Pierce A. Delta-lactoferrin induces cell death via the mitochondrial death signaling pathway by upregulating bax expression. Biometals. 2014;27:875–89. 24824995 10.1007/s10534-014-9744-5

[138] Lee YK, Thomas SN, Yang AJ, Ann DK. Doxorubicin down-regulates Kruppel-associated box domain-associated protein 1 sumoylation that relieves its transcription repression on p21WAF1/CIP1 in breast cancer MCF-7 cells. J Biol Chem. 2007;282:1595–606. 17079232 10.1074/jbc.M606306200

[139] Yang SH, Sharrocks AD. Ubc9 acetylation: a new route for achieving specificity in substrate SUMOylation. EMBO J. 2013;32:773–4. 23395903 10.1038/emboj.2013.21 PMC3604717

[140] Hsieh YL, Kuo HY, Chang CC, Naik MT, Liao PH, Ho CC, et al. Ubc9 acetylation modulates distinct SUMO target modification and hypoxia response. EMBO J. 2013;32:791–804. 23395904 10.1038/emboj.2013.5 PMC3604730

[141] Han X, Niu J, Zhao Y, Kong Q, Tong T, Han L. HDAC4 stabilizes SIRT1 via sumoylation SIRT1 to delay cellular senescence. Clin Exp Pharmacol Physiol. 2016;43:41–6. 26414199 10.1111/1440-1681.12496

[142] Yang Q, Tang J, Xu C, Zhao H, Zhou Y, Wang Y, et al. Histone deacetylase 4 inhibits NF-kappaB activation by facilitating IkappaBalpha sumoylation. J Mol Cell Biol. 2020;12:933–45. 32770227 10.1093/jmcb/mjaa043 PMC7948076

[143] Yang Y, Tse AK, Li P, Ma Q, Xiang S, Nicosia SV, et al. Inhibition of androgen receptor activity by histone deacetylase 4 through receptor SUMOylation. Oncogene. 2011;30:2207–18. 21242980 10.1038/onc.2010.600 PMC3093431

[144] Dehennaut V, Loison I, Dubuissez M, Nassour J, Abbadie C, Leprince D. DNA double-strand breaks lead to activation of hypermethylated in cancer 1 (HIC1) by SUMOylation to regulate DNA repair. J Biol Chem. 2013;288:10254–64. 23417673 10.1074/jbc.M112.421610 PMC3624409

[145] Liu W, Zeng M, Fu N. Functions of nuclear receptors SUMOylation. Clin Chim Acta. 2021;516:27–33. 33476589 10.1016/j.cca.2021.01.007

[146] Huang J, Perez-Burgos L, Placek BJ, Sengupta R, Richter M, Dorsey JA, et al. Repression of p53 activity by Smyd2-mediated methylation. Nature. 2006;444:629–32. 17108971 10.1038/nature05287

[147] Shi X, Kachirskaia I, Yamaguchi H, West LE, Wen H, Wang EW, et al. Modulation of p53 function by SET8-mediated methylation at lysine 382. Mol Cell. 2007;27:636–46. 17707234 10.1016/j.molcel.2007.07.012 PMC2693209

[148] Spektor TM, Congdon LM, Veerappan CS, Rice JC. The UBC9 E2 SUMO conjugating enzyme binds the PR-Set7 histone methyltransferase to facilitate target gene repression. PLoS ONE. 2011;6: e22785. 21829513 10.1371/journal.pone.0022785 PMC3146489

[149] Wang Q, Zhong W, Deng L, Lin Q, Lin Y, Liu H, et al. The Expression and prognostic value of SUMO1-activating enzyme subunit 1 and its potential mechanism in triple-negative breast cancer. Front Cell Dev Biol. 2021;9: 729211. 34621746 10.3389/fcell.2021.729211 PMC8490707

[150] Yang Y, Liang Z, Xia Z, Wang X, Ma Y, Sheng Z, et al. SAE1 promotes human glioma progression through activating AKT SUMOylation-mediated signaling pathways. Cell Commun Signal. 2019;17:82. 31345225 10.1186/s12964-019-0392-9 PMC6659289

[151] Fang H, Wu W, Wu Z. miR-382-3p downregulation contributes to the carcinogenesis of lung adenocarcinoma by promoting AKT SUMOylation and phosphorylation. Exp Ther Med. 2022;24:440. 35720620 10.3892/etm.2022.11367 PMC9185802

[152] Xu Y, Zuo Y, Zhang H, Kang X, Yue F, Yi Z, et al. Induction of SENP1 in endothelial cells contributes to hypoxia-driven VEGF expression and angiogenesis. J Biol Chem. 2010;285:36682–8. 20841360 10.1074/jbc.M110.164236 PMC2978597

[153] Cui CP, Wong CC, Kai AK, Ho DW, Lau EY, Tsui YM, et al. SENP1 promotes hypoxia-induced cancer stemness by HIF-1alpha deSUMOylation and SENP1/HIF-1alpha positive feedback loop. Gut. 2017;66:2149–59. 28258134 10.1136/gutjnl-2016-313264 PMC5749365

[154] Wang X, Liang X, Liang H, Wang B. SENP1/HIF-1alpha feedback loop modulates hypoxia-induced cell proliferation, invasion, and EMT in human osteosarcoma cells. J Cell Biochem. 2018;119:1819–26. 28796315 10.1002/jcb.26342

[155] Harrison H, Pegg HJ, Thompson J, Bates C, Shore P. HIF1-alpha expressing cells induce a hypoxic-like response in neighbouring cancer cells. BMC Cancer. 2018;18:674. 29925335 10.1186/s12885-018-4577-1 PMC6011406

[156] de Heer EC, Jalving M, Harris AL. HIFs, angiogenesis, and metabolism: elusive enemies in breast cancer. J Clin Invest. 2020;130:5074–87. 32870818 10.1172/JCI137552 PMC7524491

[157] Ebright RY, Zachariah MA, Micalizzi DS, Wittner BS, Niederhoffer KL, Nieman LT, et al. HIF1A signaling selectively supports proliferation of breast cancer in the brain. Nat Commun. 2020;11:6311. 33298946 10.1038/s41467-020-20144-w PMC7725834

[158] Burger AM, Gao Y, Amemiya Y, Kahn HJ, Kitching R, Yang Y, et al. A novel RING-type ubiquitin ligase breast cancer-associated gene 2 correlates with outcome in invasive breast cancer. Cancer Res. 2005;65:10401–12. 16288031 10.1158/0008-5472.CAN-05-2103

[159] Haugsten EM, Malecki J, Bjorklund SM, Olsnes S, Wesche J. Ubiquitination of fibroblast growth factor receptor 1 is required for its intracellular sorting but not for its endocytosis. Mol Biol Cell. 2008;19:3390–403. 18480409 10.1091/mbc.e07-12-1219 PMC2488279

[160] Zhang Q, Wu J, Wu R, Ma J, Du G, Jiao R, et al. DJ-1 promotes the proteasomal degradation of Fis1: implications of DJ-1 in neuronal protection. Biochem J. 2012;447:261–9. 22871147 10.1042/BJ20120598

[161] Yu Y, Peng XD, Qian XJ, Zhang KM, Huang X, Chen YH, et al. Fis1 phosphorylation by Met promotes mitochondrial fission and hepatocellular carcinoma metastasis. Signal Transduct Target Ther. 2021;6:401. 34848680 10.1038/s41392-021-00790-2 PMC8632923

[162] Gomarasca M, Lombardi G, Maroni P. SUMOylation and NEDDylation in primary and metastatic cancers to bone. Front Cell Dev Biol. 2022;10: 889002. 35465332 10.3389/fcell.2022.889002 PMC9020829

[163] Xirodimas DP, Saville MK, Bourdon JC, Hay RT, Lane DP. Mdm2-mediated NEDD8 conjugation of p53 inhibits its transcriptional activity. Cell. 2004;118:83–97. 15242646 10.1016/j.cell.2004.06.016

[164] Xie P, Zhang M, He S, Lu K, Chen Y, Xing G, et al. The covalent modifier Nedd8 is critical for the activation of Smurf1 ubiquitin ligase in tumorigenesis. Nat Commun. 2014;5:3733. 24821572 10.1038/ncomms4733

[165] Xie P, Peng Z, Chen Y, Li H, Du M, Tan Y, et al. Neddylation of PTEN regulates its nuclear import and promotes tumor development. Cell Res. 2021;31:291–311. 33299139 10.1038/s41422-020-00443-z PMC8027835

[166] Abidi N, Xirodimas DP. Regulation of cancer-related pathways by protein NEDDylation and strategies for the use of NEDD8 inhibitors in the clinic. Endocr Relat Cancer. 2015;22:T55-70. 25504797 10.1530/ERC-14-0315

[167] Zhou Q, Zheng Y, Sun Y. Neddylation regulation of mitochondrial structure and functions. Cell Biosci. 2021;11:55. 33731189 10.1186/s13578-021-00569-6 PMC7968265

[168] Gao F, Cheng J, Shi T, Yeh ET. Neddylation of a breast cancer-associated protein recruits a class III histone deacetylase that represses NFkappaB-dependent transcription. Nat Cell Biol. 2006;8:1171–7. 16998474 10.1038/ncb1483

[169] Zuo W, Huang F, Chiang YJ, Li M, Du J, Ding Y, et al. c-Cbl-mediated neddylation antagonizes ubiquitination and degradation of the TGF-beta type II receptor. Mol Cell. 2013;49:499–510. 23290524 10.1016/j.molcel.2012.12.002

[170] El Motiam A, Vidal S, de la Cruz-Herrera CF, Da Silva-Alvarez S, Baz-Martinez M, Seoane R, et al. Interplay between SUMOylation and NEDDylation regulates RPL11 localization and function. FASEB J. 2019;33:643–51. 30024791 10.1096/fj.201800341RR

[171] Babina IS, McSherry EA, Donatello S, Hill AD, Hopkins AM. A novel mechanism of regulating breast cancer cell migration via palmitoylation-dependent alterations in the lipid raft affiliation of CD44. Breast Cancer Res. 2014;16:R19. 24512624 10.1186/bcr3614 PMC3978828

[172] Fukuda I, Ito A, Hirai G, Nishimura S, Kawasaki H, Saitoh H, et al. Ginkgolic acid inhibits protein SUMOylation by blocking formation of the E1-SUMO intermediate. Chem Biol. 2009;16:133–40. 19246003 10.1016/j.chembiol.2009.01.009

[173] Hirohama M, Kumar A, Fukuda I, Matsuoka S, Igarashi Y, Saitoh H, et al. Spectomycin B1 as a novel SUMOylation inhibitor that directly binds to SUMO E2. ACS Chem Biol. 2013;8:2635–42. 24143955 10.1021/cb400630z

[174] Floris A, Mazarei M, Yang X, Robinson AE, Zhou J, Barberis A, et al. SUMOylation protects FASN against proteasomal degradation in breast cancer cells treated with grape leaf extract. Biomolecules. 2020;10. 10.3390/biom10040529 PMC7226518 32244364

[175] Kim YS, Keyser SG, Schneekloth JS Jr. Synthesis of 2’,3’,4’-trihydroxyflavone (2–D08), an inhibitor of protein sumoylation. Bioorg Med Chem Lett. 2014;24:1094–7. 24468414 10.1016/j.bmcl.2014.01.010 PMC3970184

[176] Castillo-Lluva S, Tatham MH, Jones RC, Jaffray EG, Edmondson RD, Hay RT, et al. SUMOylation of the GTPase Rac1 is required for optimal cell migration. Nat Cell Biol. 2010;12:1078–85. 20935639 10.1038/ncb2112 PMC2992316

[177] Lorente M, Garcia-Casas A, Salvador N, Martinez-Lopez A, Gabicagogeascoa E, Velasco G, et al. Inhibiting SUMO1-mediated SUMOylation induces autophagy-mediated cancer cell death and reduces tumour cell invasion via RAC1. J Cell Sci. 2019. 10.1242/jcs.234120. 10.1242/jcs.234120 PMC6826015 31578236

[178] Li R, Wei J, Jiang C, Liu D, Deng L, Zhang K, et al. Akt SUMOylation regulates cell proliferation and tumorigenesis. Cancer Res. 2013;73:5742–53. 23884910 10.1158/0008-5472.CAN-13-0538

